# Functional characterisation of components in two *Plasmodium falciparum* Cullin-RING-Ligase complexes

**DOI:** 10.1038/s41598-025-05342-0

**Published:** 2025-07-01

**Authors:** Danushka Marapana, Simon A. Cobbold, Michal Pasternak, Gerald J. Shami, Stuart A. Ralph, Sash Lopaticki, Jumana Yousef, Vineet Vaibhav, Laura F. Dagley, David Komander, Alan F. Cowman

**Affiliations:** 1 The Walter and Eliza Hall Institute of Medical Research, Parkville, 3052 Australia; 2 Department of Medical Biology, The University of Melbourne, Melbourne, 3010 Australia; 3 Department of Biochemistry and Pharmacology, Bio21 Molecular Science and Biotechnology Institute, The University of Melbourne, Melbourne, 3010 Australia; 4 School of Medical Sciences (Molecular and Cellular Biomedicine) & Australian Centre for Microscopy and Microanalysis, The University of Sydney, Sydney, NSW 2006 Australia; 5 Department of Infectious Diseases, Doherty Institute, University of Melbourne, Parkville, 3010 Australia

**Keywords:** Malaria, E3 ligase, Cullin-ring-ligase, *P. falciparum*, Ubiquitination, Biochemistry, Microbiology

## Abstract

Ubiquitination is the key eukaryotic post-translational modification that governs protein degradation, localisation, and activity which is mediated by a concerted enzyme cascade. The largest superfamily of these enzymes include the Cullin-RING-Ligase (CRL) complexes. *Plasmodium falciparum*, the causative agent of the most severe form of malaria in humans, encodes the critical proteins required for ubiquitination, but we do not yet understand the function of this pathway. Here the *P. falciparum* CRL complexes were characterised to reveal an essential but minimal repertoire controlled by two Cullin scaffolds. A PfCullin1-linked CRL complex, recruiting a single substrate receptor, was identified as being required for parasite inner-membrane biogenesis and DNA replication. A second CRL complex functioning through a PfCullin4 scaffold was identified that utilised a previously unidentified adaptor protein and receptors to support DNA replication. These results show that the *P. falciparum* CRL complexes are essential in both nuclear maintenance and membrane integrity.

**Supplementary Information:**

The online version contains supplementary material available at 10.1038/s41598-025-05342-0.

## Introduction

Malaria in humans is caused by six species of the eukaryotic protist parasite *Plasmodium* and is responsible for over 597,000 deaths in 2024^1^. The complex life cycle of the parasite, which uses a mosquito vector to transfer parasites to and from a human host, and the rapid evolution of parasite-derived drug resistance are obstacles for the malaria elimination and eradication agenda. Many of the best-in-class antimalarials target parasite proteins expressed in the asexual blood stages of the parasite, the stage responsible for the disease symptoms of this infection (reviewed in^2^). These proteins are localised in multiple compartments within the parasite and the infected erythrocyte, and their homeostasis, including protein synthesis, activity and degradation are critical for parasite survival within the human host.

In eukaryotic cells, ubiquitination plays a crucial role in mediating protein homeostasis (reviewed in^3^). Ubiquitination is the transfer of a 76-amino acid ubiquitin molecule to substrates. The transferred ubiquitin molecule can be further ubiquitinated on one or more of its seven lysine residues to produce linear or branched ubiquitin chains (reviewed in^4^). The ubiquitin chain type of a substrate ultimately signals its potential fate, thereby regulating protein levels within the cell (reviewed in^3^). Ubiquitination controls protein stability, activity, and binding to its interactome and functions in targeting substrates to the cellular proteasome for degradation, cell signalling, protein localisation, apoptosis, control of immune responses and DNA repair (reviewed in^5^).

Protein ubiquitination requires a three-step protein cascade involving ubiquitin activating E1,

ubiquitin conjugating E2 and ubiquitin ligating E3 enzymes. E3 ligases determine the specificity of substrate binding and the linkage which ubiquitin chains arise on the target protein (reviewed in^6^). Three varieties of E3 ligases exist within eukaryotic cells, including the **R**eally **I**nteresting **N**ew **G**ene (RING), **H**omologous to **E**6AP **c**arboxy **t**erminus (HECT) and **RING**-**B**etween-**RING** (RBR) type enzymes. The majority of E3 ligases belong to the RING family and of those, the Cullin-RING E3 ubiquitin ligases (CRL) are the largest superfamily of E3 ubiquitin ligases (reviewed in^7^). CRLs contain over 200 members in humans and facilitate nearly 20% of all ubiquitination events in cells^8^reviewed in^9^). CRLs function in many diverse processes of cells including cell division, response to hypoxia, cell growth and DNA damage repair. Mutations within the CRL subunits are causative of multiple human diseases such as cancers, myopathies, and diabetes (reviewed in^7^).

All CRLs consist of a scaffold Cullin protein, of which there are at least 7 types (reviewed in^9^). A RING-type protein, either RBX1 or RBX2 binds the C-terminus of the Cullin protein, and a substrate adaptor protein, including SKP1, DDB1 and Elongin B-C, interacts with the Cullin N-terminus. RBX1/RBX2 binds Ubiquitin-bound E2 enzymes and creates an activated E3 ubiquitin ligase complex. The adaptor proteins recruit substrate receptors to the complex and thereby promote substrate diversity. Human cells contain up to 600 different substrate receptors containing either F-box, BTB or SOCS/BC-box domains (reviewed in^10^**)**. For instance, in human cells up to 70 different FBOX receptors can be interchanged on a single Cullin1-SKP1 based CRL complex^11,12^. It is this expansion of substrate receptors which leads to the exquisite specificity of distinct CRL complexes to their associated substrates.

Although the essential enzymes and proteins required for ubiquitination can be identified through homology^13^direct evidence of ubiquitinated proteins in *Plasmodium* spp. has only recently been demonstrated^14,15^. Analysis of di-Gly remnant peptides arising from ubiquitination identified 1461 distinct ubiquitination sites from 546 parasite proteins^15^. While this study conclusively identified the incidence and importance of ubiquitination of *P. falciparum* proteins, there are no definitive data yet on the identity of the E3 ubiquitin ligases that mediate the process.

In this study, we characterized the CRL E3 ubiquitin ligases of the causative agent of the most lethal form of malaria in humans, *P. falciparum*. A minimal group of CRL components in asexual blood stage parasites was identified, controlled by two Cullin scaffolds. A PfCullin1-linked SCF complex was identified, which is involved in the biogenesis of the inner membrane complex (IMC) and DNA replication. A second CRL complex functioning through a PfCullin4 scaffold utilises a previously unidentified adaptor protein PfCPSFA (*P. falciparum* *C*leavage and *P*olyadenylation *S*pecific *F*actor Subunit *A*). This second CRL complex appears to be required for DNA replication. These results demonstrate the critical function of parasite expressed E3 ubiquitin ligases in malaria of humans.

## Materials and methods

### Parasite culture

*P. falciparum* asexual blood stage parasite cultures were grown in in vitro culture^16^. All parasites were supplied with O + erythrocyte (Australian Red Cross Bloodbank, South Melbourne, Australia) at 4% hematocrit in Roswell Park Memorial Institute (RPMI) 1640 medium supplemented with 26 mM 4-(2-hydroxyethyl)piperazine-1-ethanesulfonic acid (HEPES), 50 mg/mL hypoxanthine, 20 mg/mL gentamicin, 2.9% NaHCO3 and 10% Albumax IITM (GIBCO). Cultures were incubated at 37 °C in a gaseous mix of 94% N_2_, 1% O_2_ and 5% CO_2_.

### Parasite transfection

A combined linearized homology directed repair (HDR) plasmid (100 µg) and circular guide plasmid (100 µg) were resuspended in 50ul Tris-EDTA buffer (Sigma) and transfected into synchronised schizonts suspended in with 85 µl Solution 1 and 15 µl Solution 2 of Basic Parasite Nucleofector Kit 2. Program UL-33 with the Amaxa Nucleofector 2b (Lonza) was used. Parasites with an integrated hDHFR or BSD drug-resistance cassette were selected and maintained on 2.5 nM WR99210 (Jacobus Pharmaceuticals) or 2.5 µg/ml Blasticidin S (Sigma) respectively. When DiCre-mediated gene excision was required, ring-stage parasites were treated with 10 nM rapamycin dissolved in 0.0004% DMSO.

## Parasite cell line generation

### PfSKP1-HA

For tagging and conditional deletion of PfSKP1 (accession PF3D7_1367000), a 3’ homology region was amplified from 3D7 gDNA with oligos DM449/DM450. The amplicon was cloned into p1.2_RON3-mNeonGreen vector^17^ by the restriction sites *Eco*RI/*Kas*I to create p1.2_RON3-mNeonGreen_SKP1_3’ vector. The 5’ homology and codon optimized PfSKP1 gene region containing LoxP sites was produced by GenScript Biotech and cloned into p1.2_RON3-mNeonGreen_SKP1_3’ with *NotI*/*SacII* restriction sites to produce p1.2_SKP1-HA vector. Guide oligos DM447/DM448 designed to induce a double stranded break in PfSKP1 at genomic position 553 were generated using InFusion (Takara) and cloned into pUF1-Cas9G using previously published methods^18^.

### PfRBX1-HA

For tagging and conditional deletion of PfRBX1 (accession PF3D7_0319100), a 3’ homology region was amplified from 3D7 gDNA with oligos DM539/DM540. The amplicon was cloned into p1.2_RON3-mNeonGreen vector^17^ by the restriction sites *Eco* RI/*Kas* I to create p1.2_RON3-mNeonGreen_RBX1_3’ vector. The 5’ homology and codon optimized PfRBX1 gene region containing LoxP sites was produced by GenScript Biotech and cloned into p1.2_RON3-mNeonGreen_RBX1_3’ with *Not*I/*SacII* restriction sites to produce p1.2_RBX1-HA vector. Guide oligos DM541/DM542 designed to induce a double stranded break in PfRBX1 at genomic position 66 were InFusion cloned into pUF1-Cas9G using previously published methods^18^.

### PfFBOX01-HA

For tagging and conditional deletion of PfFBOX01 (accession PF3D7_0619700), a 3’ homology region was amplified from 3D7 gDNA with oligos DM550/DM551. The amplicon was cloned into p1.2_RON3-mNeonGreen vector^17^ by the restriction sites *Eco* RI/*Kas* I to create p1.2_RON3-mNeonGreen_ FBOX01_3’ vector. The 5’ homology and codon optimized PfFBOX01 gene region containing LoxP sites was produced by GenScript Biotech and cloned into p1.2_RON3-mNeonGreen_FBOX01_3’ with *Not*I/*SacII* restriction sites to produce p1.2_ PfFBOX01-HA vector. Guide oligos DM552/DM553 designed to induce a double stranded break in PfFBOX01 at genomic position 742 were generated using InFusion (Takara) and cloned into pUF1-Cas9G as described^18^.

### PfCPSF-HA

For tagging and conditional deletion of PfCPSF-A (accession PF3D7_0317700), a 3’ homology region was amplified from 3D7 gDNA with oligos DM707/DM708. The amplicon was cloned into p1.2_RON3-mNeonGreen^17^ vector by the restriction sites *EcoR*I/*Kas*I to create p1.2_RON3-mNeonGreen_ CPSF-A _3’ vector. The 5’ homology and codon optimized PfCPSF-A gene region containing LoxP sites was produced by GenScript Biotech and cloned into p1.2_RON3-mNeonGreen_ CPSF-A _3’ with *NotI/SacII* restriction sites to produce p1.2_ PfCPSF-A-HA vector. Guide oligos DM705/DM706 designed to induce a double stranded break in PfCPSF-A at genomic position 6569 were generated using InFusion (Takara) and cloned into pUF1-Cas9G using previously published methods^18^.

### PfMCM4-Flag-glmS

For tagging of PfMCM4 (accession PF3D7_1317100), a 3’ homology region was amplified from 3D7 gDNA with oligos DM595/DM596. The amplicon was cloned into pFGB1_BSD vector^19^ by the restriction sites *EcoR*I/*Pst*I to create p1.2_ FGB1_MCM4 _3’ vector. The 5’ homology and codon optimized PfMCM4-Flag-glmS_gene region containing *LoxP* sites was produced by Integrated DNA Technologies (IDT) and cloned into p1.2_ FGB1_MCM4 _3’ with *Not*I/*XhoI* restriction sites to produce p1.2_ MCM4-Flag-glmS vector. Guide oligos DM593/DM594 designed to induce a double stranded break in PfMCM4 at genomic position 3573 were generated using InFusion (Takara) and cloned into pUF1-Cas9G using previously published methods^18^.

### PfPP7-Flag-glmS

For tagging of PfPP7 (accession PF3D7_1423300), a 3’ homology region was amplified from 3D7 gDNA with oligos DM651/DM652. The amplicon was cloned into pFGB1_BSD vector^19^ by the restriction sites *EcoR*I/*Pst*I to create p1.2_ FGB1_PP7 _3’ vector. The 5’ homology and codon optimized PfPP7-Flag-glmS_gene region containing LoxP sites was produced by GenScript Biotech and cloned into p1.2_ FGB1_PP7 _3’ with *Not*I/*XhoI* restriction sites to produce p1.2_ PP7-Flag-glmS vector. Guide oligos DM653/DM654 designed to induce a double stranded break in PfPP7 at genomic position 5289 were generated using InFusion (Takara) and cloned into pUF1-Cas9G using previously published methods^18^.

### Pf0903600-Flag-glmS

For tagging of PfPP7 (accession PF3D7_1423300), a 3’ homology region was amplified from 3D7 gDNA with oligos DM655/DM656. The amplicon was cloned into pFGB1_BSD vector^19^ by the restriction sites *EcoR*I/*Pst*I to create p1.2_ FGB1_0903600 _3’ vector. The 5’ homology and codon optimized 0903600-Flag-glmS_gene region containing LoxP sites was produced by GenScript Biotech and cloned into p1.2_ FGB1_PP7 _3’ with *Not*I/*XhoI* restriction sites to produce p1.2_ 0903600-Flag-glmS vector. Guide oligos DM657/DM658 designed to induce a double stranded break in Pf0903600 at genomic position 4189 were generated using InFusion (Takara) and cloned into pUF1-Cas9G using previously published methods^18^.

### Immunoprecipitation, western blotting and antibodies

Saponin pellets of 5 × 30 ml schizont stage parasite cultures were incubated with 0.5X RIPA buffer (0.5% deoxycholate, 75 mM NaCl, 0.05% SDS and 1% TX-100) supplemented with Complete Protease Inhibitors (Roche) and sonicated gently at 15% amplitude for 3 × 30 s on ice. Proteins were extracted overnight at 4 °C and clarified supernatant was immunoprecipitated with anti-HA agarose (Merck; catalogue number 11815016001) or anti-Flag agarose (Merck; catalogue number A2220) antibodies for 1 h at room temperature. In all cases, beads were thoroughly washed with 0.5X RIPA buffer and water before proteins were eluted using either hot 0.5% SDS or 4X Laemmli’s sample buffer for mass spectrometry or western blotting respectively. When required, elutes were then reducing by addition of 2-mercaptoethanol and boiled at 95 °C for 5 min.

Proteins were separated on 4–12% Bis-Tris reducing polyacrylamide gels (Life Technologies) and transferred to nitrocellulose. Membranes were blocked for 1 h with 5% Skim Milk in PBS + 0.1% Tween-20. The following antibodies and dilutions were used for western blotting: Rat anti-HA peroxidase 1:1000 (Merck; catalogue number 12013819001), Mouse anti-Flag peroxidase 1:1000 (Merck; Catalogue number A8592), Mouse anti-Ubiquitin PD41 1:1000 (Cell Signalling; catalogue number 3936) Rabbit anti-Aldolase 1:2000 (WEHI Antibody Facility).

### Parasite growth assays

Following rapamycin treatment, parasites were stained for 15 min with 1:1,000 Ethidium Bromide (Merck; catalogue number 09-0617) in HTPBS. At least 100,000 erythrocytes were analysed using a LSR II Flow cytometer (Becton Dickinson) and assessed for the presence of parasites.

### Live cell imaging and immunofluorescence (IFA) imaging

For fixed imaging, intra-erythrocytic parasites were fixed with 4% Paraformaldehyde and 0.0075% Glutaraldehyde for 30 min, permeabilised with 0.1% TX-100 in HTPBS for 15 min and incubated in blocking solution (3% BSA in PBS) for 1 h. SPY505 dye (Spirochrome; catalogue number SC101), primary antibodies Rat anti-HA 1:100 (Merck; catalogue number 11867423001), rabbit anti-PfGAP45 1:1000 (WEHI Antibody Facility), mouse anti-Flag 1:1000 (Merck; catalogue number F1804), rabbit anti- ERC (1:1000), rabbit anti-histone PfH2AZ (1:1000) were used. The following secondary Alexa fluorophores were used at 1:300 dilution. Donkey anti-rat 488 (catalogue number A21208, lot 2310102), chicken anti-rabbit 594 (catalogue number A21442, lot 2110863), goat anti-mouse 488 (catalogue number A11001).

Images were viewed on a Deltavision Elite microscope and collected with a Coolsnap HQ2 CCD camera through an Olympus 100x UPlanSApo NA1.4 objective with SoftWorx software. Images were assembled with ImageJ2 Fiji 1.54f and Adobe Photoshop CS6.

For Airyscan imaging, Z-stacks of fluorescently labelled infected red blood cells were imaged with Zeiss 880 inverted or Zeiss 880 upright microscope equipped with a Plan Apochromat 63x/1.4 oil objective and an Airyscan detector. Images were assembled with ImageJ2 Fiji 1.54f and Adobe Photoshop CS6.

### Mass spectrometry analysis

#### Sample preparation

Eluates from immunoprecipitation were prepared for LC-MS/MS analysis using the Filter Assisted Sample Preparation (FASP) method^20^. Briefly, eluates were added to a Vivacon^®^ 30 kDa MWCO (Sartorius), and proteins were solubilised and reduced in 6 M Urea containing 10 mM TCEP for 30 min, or for 60 min where cell lysates had been treated with 0.5mM DSP cross-linker prior to immunoprecipitation. Alkylation was performed after spins and washes with 50 mM Iodoacetamide in Urea, and then buffer exchange was performed from Urea to ammonium bicarbonate (AmBic) prior to digestion using 1 mg Trypsin (SoLuTrypsin, Sigma Aldrich) overnight at 37 °C. Peptides were then eluted in AmBic, digest neutralised to 1% formic acid (FA), and then the peptides lyophilised to dryness in a vacuum centrifuge (LabConco). Peptides we reconstituted in 2% acetonitrile (ACN), 0.1% FA for LC-MS/MS analysis.

For global CPSFA proteomics, cell pellets were lysed in 200 µl of preheated (95 °C) buffer (2.5% SDS in 100 mM Tris-HCl, pH 8.5). DNA was hydrolysed with the addition of 2 µl neat TFA (Sigma) and lysates were neutralised to pH 8.5 by addition of 1 M Tris-HCl as previously described^21^. Protein concentration was determined using Pierce™ BCA Protein Assay Kit following manufacturers’ instructions. Cell lysates (20 µg protein per replicate) were transferred to 0.5 ml LoBind Deep Well plate (Eppendorf) prepared for mass spectrometry analysis using the modified SP3 protocol^22^with some modifications. Briefly, samples were subjected to simultaneous reduction and alkylation with a final concentration of 10 mM Tris (2-carboxyethyl) phosphine (TCEP) and 40 mM 2-chloracetamide followed by heating at 95 °C for 10 min. Prewashed magnetic PureCube Carboxy agarose beads (20 µl, Cube Biotech) were added to all the samples along with acetonitrile (ACN,70% v/v final concentration) and incubated at room temperature for 20 min. Samples were placed on a magnetic rack and supernatants were discarded, and beads were washed twice with 70% ethanol and once with neat ACN. ACN was completely evaporated from the tubes using a CentriVap (Labconco) before the addition of digestion buffer (50 mM Tris-HCl, pH 8) containing 1 µg each of enzymes Lys-C (Wako, 129–02541) and SOLu-Trypsin (Sigma-Aldrich, EMS0004). Trypsin-LysC on-bead digestion was performed with agitation (400 rpm) for 1 h at 37 °C on a ThermoMixer C (Eppendorf). Following digestion, the samples were transferred to pre-equilibrated C18 StageTips (2× plugs of 3 M Empore resin, no. 2215) for sample clean-up. The eluates were lyophilized to dryness before being reconstituted in 50 µL 0.1% FA/2% ACN ready for mass spectrometry analysis.

#### Data-independent acquisition (DIA)-based mass spectrometry analysis

LC-MS/MS analysis of interacting proteins from the immunoprecipitation and whole cell lysates were performed on a timsTOF Pro MS (Bruker) with a CaptiveSpray source. For DIA analysis, peptides (1 µL) were separated on a C_18_ fused silica column (inner diameter 75 μm, OD 360 μm × 15 cm length, 1.6 μm C18 beads) packed into an emitter tip (Aurora, IonOpticks) using a custom nano-flow HPLC system (Thermo Ultimate 300 RSLC Nano-LC, PAL systems CTC autosampler). Peptides were loaded directly onto the column at a constant flow rate of 600 nL/min with buffer A (99.9% Milli-Q water, 0.1% FA) and eluted with a 30-min linear gradient at 400 nL/min from 2 to 34% buffer B (90% ACN, 0.1% FA). The timsTOF Pro MS was operated in diaPASEF mode using Compass Hystar 5.1. The settings on the TIMS analyzer were as follows: Lock Duty Cycle to 100% with equal accumulation and ramp times of 100 ms, and 1/K0 Start 0.6 V.·/cm^2^ End 1.6 V·s/cm^2^, Capillary Voltage 1400 V, Dry Gas 3 l/min, Dry Temp 180 °C. The DIA methods were set up using timsTOF control (v2.0.18.0) for two windows in each diaPASEF scan, with window placement overlapping the diagonal scan line for doubly and triply charged peptides in the m/z – ion mobility plane across 16 × 25 m/z precursor isolation windows (resulting in 32 windows) defined from *m/z* 400 to 1,200, with 1 Da overlap, and CID collision energy ramped stepwise from 20 eV at 0.8 V·s/cm^2^ to 59 eV at 1.3 V·s/cm^2^.

For the Data Dependent Analysis (DDA) analysis of potential PTMs regulating MCM4, the RBX1-HA and MCM4-FLAG eluates, SKP1-HA and MCM4-FLAG and controls were analysed in DDA on an Orbitrap Eclipse Tribrid mass spectrometer interfaced with Neo Vanquish liquid chromatography system (Thermo Fisher Scientific). Peptides were loaded using pressure-controlled loading with a maximum pressure of 1,500 bar on a 15 cm length Aurora C_18_ fused silica column as described above (IonOpticks), using Easy nLC source and electro sprayed directly into the mass spectrometer and eluted with a 30-min gradient at 400 nL/min from 3 to 30% buffer B (80% ACN, 0.1% FA) for 20 min, then 30–40% buffer B for 10 min and 35–99% buffer B for 5 min, and then maintained at 90% buffer B for 10 min before the column was equilibrated at high pressure for 2 min. MS1 spectra were acquired in the Orbitrap (*R* = 120k; normalised AGC target = standard; MaxIT = Auto; RF Lens = 30%; scan range = 350–1500; profile data). Dynamic exclusion was employed for 30 s excluding all charge states for a given precursor. Data dependent MS2 spectra were collected in the Orbitrap for precursors with charge states 2–7 (*R* = 30k; HCD collision energy mode = fixed; normalized HCD collision energies = 30%; scan range first mass = 120 m/z; normalised AGC target = 200%; MaxIT = 45 ms).

#### Database searching

DIA-NN 1.8 ^23^ was used for searching of timsTOF diaPASEF .d files in library-free mode with match between runs (MBR) enabled. Data were searched against sequences from the *Plasmodium falciparum* 3D7 proteome downloaded from Uniprot (downloaded October 2021). The search was set to trypsin specificity, peptide length of 7–30 residues, cysteine carbidomethylation was fixed, and the maximum number of missed cleavages at 2. Variable modifications included n-terminal acetylation and methionine oxidation and max variable modifications set to 1. Mass accuracy was set to 10 ppm for both MS1 and MS2 spectra and the quantification strategy to robust LC (high precision).

The DDA data was searched using MaxQuant (v1.6.17)^24^. The Uniprot database above was used for database searching alongside sequences of common contaminants, using parameters of trypsin digestion, maximum two missed cleavages, carbamidomethylation as a fixed modification, protein N-terminal acetylation, methionine oxidation and phosphorylation (STY) as variable modifications, peptide length of minimum seven amino acids, and peptide and protein false discovery rate set to 0.01.

#### Data and statistical analysis of proteomic data

For reporting precursor and protein numbers for DIA, outputs were filtered at precursor q-value < 1% and PG protein q-value < 1%, and the PG.MaxLFQ was used to obtain the normalised quantity for protein groups based on proteotypic peptides (i.e. unique proteins). Processing and analysis of co-IP dataset and phosphorylation sites were conducted using R (version 4.2.1). To ensure data integrity, false hits, including contaminants, were removed. For analysis of phosphorylation sites, only peptides with a site localization probability greater than 0.75 were retained. Furthermore, peptides that quantified in at least 60% of replicates within at least one condition were considered for further analysis.

For normalization, cyclicloess method was applied to the co-precipitated proteins identified in the DIA dataset, while RUV-IIIC^25^ package (v.1.0.19) was utilised on phosphorylation dataset. RUVIIIC incorporates negative controls and utilises replicate information to enhance the accuracy of normalization. Negative controls were selected based on empirical criteria, specifically, those with a coefficient of variation (CV%) of < 5% of the Invariant proteins across all conditions (P-value > 0.5).

Missing values were imputed using the Barycenter approach (v2-MNAR) as implemented in the msImpute package (v.1.7.0)^26^. Multivariate analysis, principal component analysis (PCA), was employed to identify any potential outliers.

Differential analysis was carried out using the limma package (v. 3.54.2). A protein was deemed significantly differentially expressed if the fold change was false discovery rate (FDR) was ≤ 5% following Benjamini–Hochberg (BH) correction.

#### Global proteomics analysis of whole cell lysates

Saponin-isolated parasite pellets were washed with PBS and then extracted with 5% SDS. Lysates were centrifuged at 14,000 g for 10 min to pellet any insoluble material and the lysate transferred to a Lo-bind Eppendorf tube (1.5 mL). A BCA assay was performed on representative samples to determine the protein concentration and 40 mg of protein per sample was processed for LC-MS analysis via the USP3 method^21^.

Following peptide recovery and SDB stage tip clean-up (GL Sciences), a pooled peptide sample was generated and fractionated offline into 88 wells using an offline LC-UV fractionation system (Dionex). 88 fractions were concatenated into 12 samples and a spectral library was generated by acquiring the 12 fractionated samples on a timsTOF Pro MS under DDA-PASEF acquisition. Peptides were separated on a 25 cm C18 column (Ion Opticks − 75 μm ID, OD 360 μm × length, 1.6 μm) using a custom nano-flow HPLC system (Thermo Ultimate 300 RSLC Nano-LC, PAL systems CTC autosampler), at 400 nL/min with buffer A (99.9% Milli-Q water, 0.1% FA) and eluted with a 90 min linear gradient from 2 to 34% buffer B (99.9% ACN, 0.1% FA). Individual samples were acquired with DIA-PASEF on the same system with a 45 min gradient. DIA-PASEF methods were adapted from^27^16 × 25 m/z precursor isolation windows scans (resulting in 32 windows) were aligned across the m/z (defined from m/z 400- to 1,200) and ion mobility (0.8–1.4) with 1 Da overlap. The TIMS ramp time was 100 ms and CID collision energy ramped stepwise from 20 eV at 0.8 V·s/cm2 to 59 eV at 1.3 V·s/cm2.

Spectral library generation was performed with MSFragger^28^ using the *P. falciparum* 3D7 Uniprot database UP000001450 and default closed search parameters. DIA data were searched with DIA-NN 1.8 ^29^using the fractionated spectral library. MS1 and mass accuracy was set to 15 ppm and the precursor FDR was set at 1%. Protein quantification was performed using the DIA-NN in-built MaxLFQ algorithm Data processing and analysis were conducted using R (version 4.2.1). Downstream data processing and analysis was performed using the DEP package in R 4.2.1 ^30^. The mass spectrometry proteomics data have been deposited to the ProteomeXchange Consortium via the PRIDE^31^ partner repository with the Project Name: Functional characterisation of Cullin-Ring-Ligases in *Plasmodium falciparum* Project accession: PXD063957.

### Electron microscopy

Schizont-stage parasites treated with DMSO, or rapamycin were purified on a 70% Percoll cushion to remove uninfected erythrocytes. Parasites were then washed with RPMI lacking serum or Albumax and fixed first in 2% glutaraldehyde, 2% formaldehyde in 0.1 M Sorensen’s Phosphate Buffer pH 7.4), postfixed in 1% osmium tetroxide and 1.5% potassium ferrocyanide, then subsequently incubated in 1% tannic acid and then in 1% uranyl acetate. Samples were then dehydrated stepwise in ethanol and then acetone and embedded in Procure 812 resin. Ultrathin (90–100 nm) sections were cut using a Leica EM UC7 Ultramicrotome. Samples were post-stained in uranyl acetate and Reynold’s lead citrate and imaged using a Tecnai F20 TEM.

### Statistics and reproducibility

All experiments were conducted with a minimum of three biologically independent replicates.

## Results

### Identification of core CRL complex components in *P. falciparum*

The annotated proteome of *P. falciparum* appears to contain minimal components of canonical Cullin-RING-Ligases^32^. These include two homologues of the seven known eukaryotic Cullin subunits PfCullin1 (PF3D7_0811000), PfCullin2 (PF3D7_0629800) (Fig. S1). The RING-domain containing PfRBX1 (PF3D7_0319100), PfNEDD8 (PF3D7_1313000) and the adaptor protein PfSKP1 (PF3D7_1367000) can also be identified with high confidence due to conservation of the critical functional domains^32^. A previous pulldown of PfNedd8 found that it interacted with both PfCullin1 and PfCullin2^33^. While other eukaryotes contain up to 70 FBOX proteins, *P. falciparum* appears to have only two that can be predicted bioinformatically; PfFBOX01 (PF3D7_0619700) and PfFBOX06 (PF3D7_0614700). Transposon mutagenesis analysis of the canonical PfSCF complex members has predicted that all protein functions are essential, except for PfRBX1^34^ (Fig. S1). However, the identified transposon insertions of PfRBX1 are not within the coding region and appear in the 5’ and 3’ untranslated regions suggesting caution with the interpretation of non-essentiality. All components are transcribed mainly in intra-erythrocytic schizont stages, suggesting at functions required for development of schizonts and merozoites (https://plasmodb.org)^35^.

**Fig. 1:**
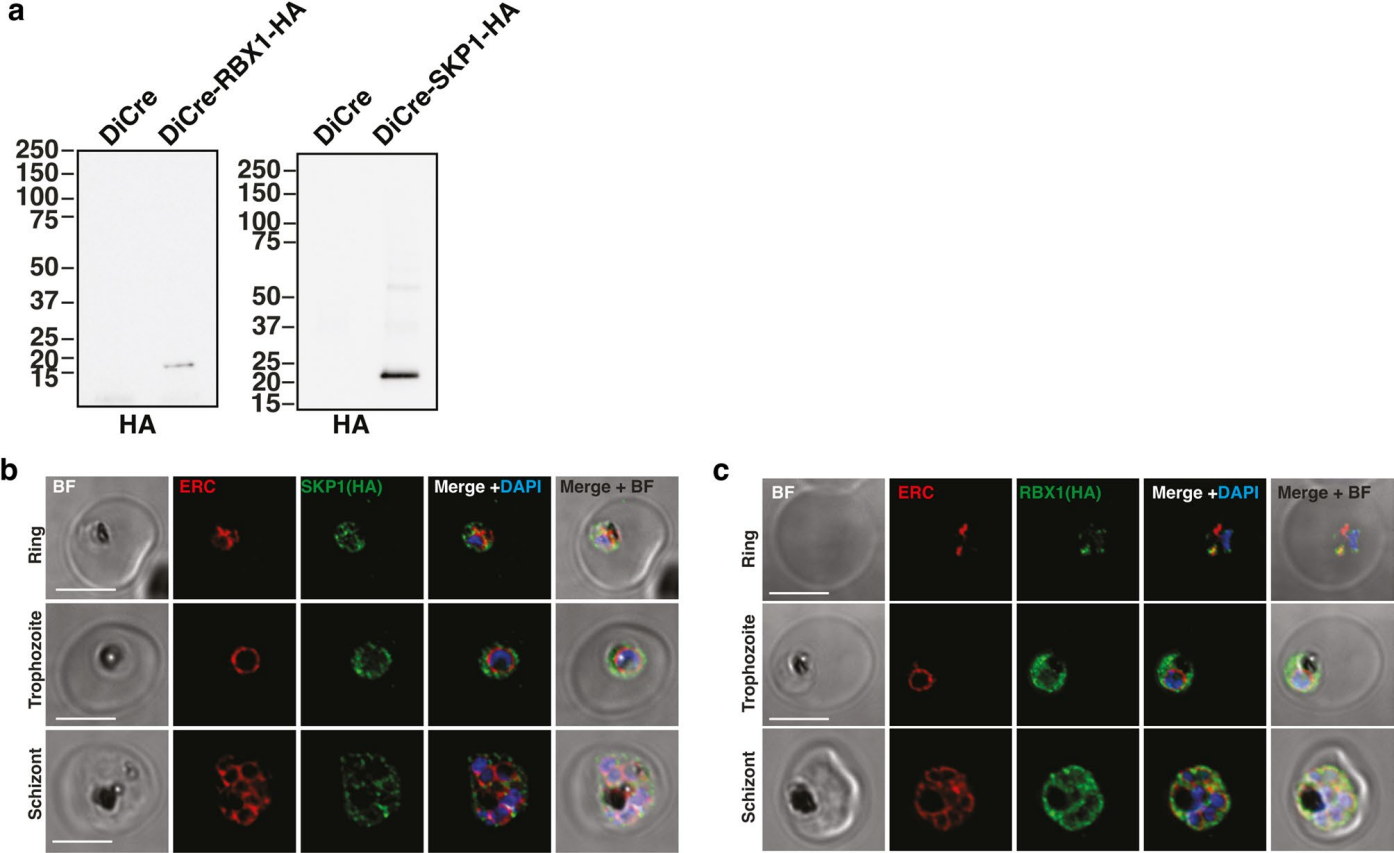
Identification of core PfCRL complex components. (**a**) Immunoblots showing C-terminal tagging of DiCre-PfRBX1-HA and DiCre-PfSKP1-HA transgenic strain parasites by anti-HA antibodies. (**b**) Immunofluorescence imaging of ring, trophozoite and schizont stages of DiCre-PfSKP1-HA parasites. PfSKP1, parasite ER membrane and DNA levels were visualised using anti-HA, anti-ERC antibodies and DAPI staining respectively. (**c**) Immunofluorescence imaging of ring, trophozoite and schizont stages of DiCre-PfRBX1-HA parasites. RBX1, parasite ER membrane and DNA levels were visualised using anti-HA, anti-ERC antibodies and DAPI staining respectively.

To better understand the functions of the PfCRL complexes, the endogenous PfRBX1 and PfSKP1 proteins were tagged with a haemaggluttinin (HA) triple peptide at the C-terminus and proteins of the expected molecular weights were detected with anti-HA antibodies in asexual blood stage parasites (Fig. 1a, Fig. S8). The same strategy was attempted with the *pfcullin* genes, but the desired transgenic parasites could not be derived, suggesting an essential role of unhindered PfCullin C-termini for parasite survival. Immunofluorescence analysis of ring, trophozoite and schizont stages showed that PfRBX1-HA and PfSKP1-HA were localised within the parasite cytoplasm compared with the parasite endoplasmic reticulum marker PfERC (Fig. 1. b, c). This localisation of PfRBX1 and PfSKP1 was typical of that observed for the protein orthologues in other species (reviewed in^36^).

### PfRBX1 and PfSKP1 are essential for development of schizont stage

**Fig. 2:**
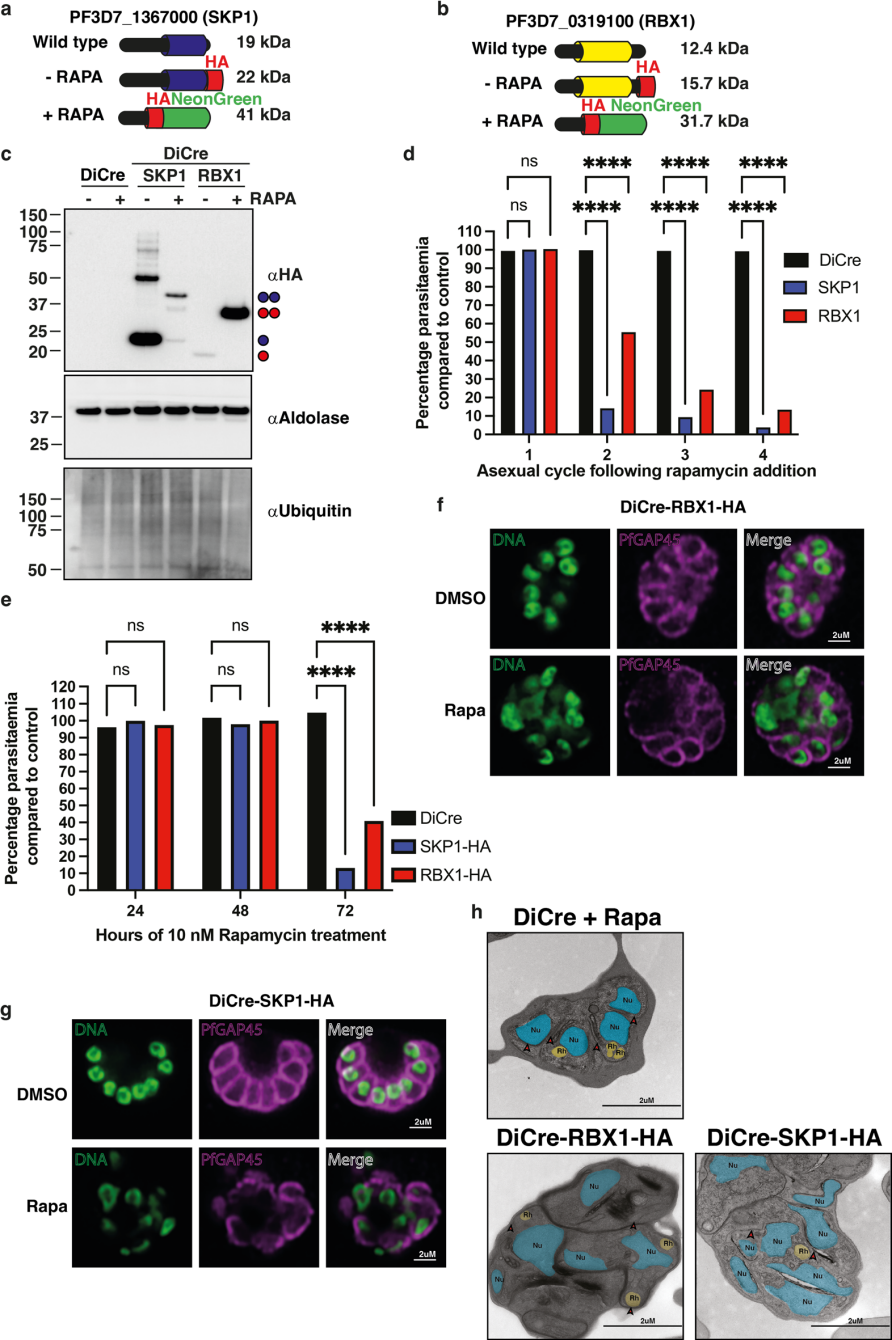
PfRBX1 and PfSKP1 are essential for intra-erythrocytic *P. falciparum* survival. (**a**) Schematic of protein size of wild type, HA-tagged and truncated PfSKP1 in DiCre-PfSKP1-HA transgenic parasite strain. FBOX-binding domain in blue, HA tag in red and Neon Green domain in green colours respectively. (**b**) Schematic of protein size of wild type, HA-tagged and truncated PfRBX1 in DiCre-PfRBX1-HA transgenic parasite strain. RING domain in yellow, HA tag in red and Neon Green domain in green colours respectively. (**c**) Immunoblots of schizont stage parental DiCre, DICre-PfSKP1-HA and DiCre-PfRBX1-HA parasites treated with 0 or 10 nM rapamycin for 48 h. DiCre-Rapamycin mediated excision, protein loading, and total ubiquitination of parasites assessed by anti-HA, anti-Aldolase, and anti-Ubiquitin antibodies respectively. Full-length and truncated PfSKP1-HA and PfRBX1-HA is denoted by one and two blue circles respectively. (**d**) Analysis of ring stage DiCre, DiCre-PfSKP1-HA and DiCre-PfRBX1-HA parasites grown on DMSO or 10 nM Rapamycin for 3 intraerythrocytic cycles. Parasitemia is assessed as a percentage compared to growth of control parasite grown on DMSO. Data are mean ± s.e.m for *n* = 3 biological replicates with 2 technical duplicates and compared by two-way Anova (Dunnett’s multiple comparison test. (**e**) Growth of ring stage DiCre, DiCre-PfSKP1-HA and DiCre-PfRBX1-HA parasites on DMSO or 10nM Rapamycin for 1.5 intraerythrocytic cycles. Parasitemia was assessed every 24 h following treatment and assessed as a percentage compared to growth of control parasite grown on DMSO. Data are mean ± s.e.m for *n* = 3 biological replicates and compared by two-way Anova (Dunnett’s multiple comparison test. (**f**) Immunofluorescence imaging of DMSO (top panels) and Rapamycin (bottom panels) treated DiCre-PfRBX1-HA schizonts. Parasite membrane integrity and DNA levels visualised using anti-PfGAP45 antibodies and SPY505 DNA dye respectively. (**g**) Immunofluorescence imaging of DMSO (top panels) and Rapamycin (bottom panels) treated DiCre-PfSKP1-HA schizonts. Parasite membrane integrity and DNA levels visualised using anti-PfGAP45 antibodies and SPY505 DNA dye respectively. (**h**) Transmission electron microscopy of Rapamycin treated parental DiCre, DiCre-PfRBX1-HA and DiCre-PfSKP1-HA schizont stage parasites. The nuclei and rhoptries have been manually annotated and coloured blue and yellow respectively using a trace feature from Adobe Illustrator that identifies these organelles.

PfRBX1-HA and PfSKP1-HA parasite lines were designed for DiCre-rapamycin mediated conditional excision of the highly conserved domains of each protein^37^. The full-length proteins were HA tagged at the C-terminus, however, upon addition of rapamycin excision was induced resulting in removal of the LoxP flanked gene sequences and sequentially C-terminally tagged the resulting protein species with a HA-NeonGreen epitope tag (Fig. 2a, b). To determine the level of gene excision, schizont stage PfSKP1-HA and PfRBX1-HA parasites treated with DMSO or rapamycin were analysed by immunoblot (Fig. 2c, Fig. S9). Near complete DiCre-mediated excision was observed in both proteins evidenced by the lack of full-length protein and the appearance of the truncated version, which resulted in a higher molecular weight species containing the HA-NeonGreen fusion. Conditional knockout (cKO) of PfRBX1 or PfSKP1 did not result in significant alterations of global ubiquitin levels within the parasite (Fig. 2c, Fig. S9). Interestingly, the PfRBX1 HA-Neon green protein fusion in the conditional knockout parasite line displayed hyperstability but given that the key domains of the native protein are lacking it is unlikely to retain any functionality.

To determine the effect of the loss of function for PfSKP1 and PfRBX1 proteins ring-stage cultures were treated with rapamycin and monitored for growth over 120 h (four blood stage life cycles) (Fig. 2d). Upon addition of rapamycin parental DiCre parasites were unaffected in growth while parasitemia of the PfSKP1-HA and PfRBX1-HA parasites decreased by 90% compared to controls. PfSKP1-HA parasites underwent a more rapid decrease in parasitemia compared to PfRBX1-HA following the first cycle of growth with an 85% decrease compared to 30% respectively. Therefore, both PfSKP1 and PfRBX1 play essential roles required for normal blood stage growth of *P. falciparum*.

The asexual life cycle stage in which parasite growth was affected due to loss of PfSKP1 and PfRBX1 function was determined by following parasite growth and stage distribution over 72 h. A reduction in parasitemia was observed at ring stage in the cycle following rapamycin addition, with a 90% reduction for PfSKP1-HA parasites compared to 60% for PfRBX1-HA (Fig. 2e). Increased proportions of schizonts were observed for rapamycin-treated PfSKP1-HA parasites at 48 h post-rapamycin addition (Fig. S2 a and b). While unaffected at 48 h, increased proportions of ring-stage parasites were observed for ring-stage PfRBX1-HA parasites 72 h following rapamycin treatment suggesting a general attenuation of parasite growth upon functional depletion of PfRBX1 (Fig. S2 a and b). This suggests the function of PfSKP1 and PFRBX1 both play essential roles in development of schizont stages blocking transition to rings in the next growth cycle.

To investigate morphological changes caused by loss of PfRBX1 and PfSKP1 function, immunofluorescence analysis was carried out on schizonts (Fig. 2f, g). Changes to DNA and inner membrane complex architecture were assessed using the DNA binding SPY505 dye and an antibody against PfGAP45, an important protein required for the development and structure of the inner membrane complex^38^ respectively. While DNA was packaged within the PfGAP45 encircled merozoite in DMSO-treated parasites, rapamycin-treated *pfrbx1* deletion resulted in schizonts with aberrant DNA morphologies with some nuclei considerably larger than others. While most parasites showed normal PfGAP45 morphologies, others had merozoites that lacked PfGAP45 encirclement. These phenotypes were more pronounced in *pfskp1* deleted parasites, where both DNA localisation and PfGAP45 distribution were severely disrupted resulting in non-viable schizonts. No significant changes were observed in the levels of distribution of the histone protein PfH2AZ^39^ as detected by immunofluorescence analysis in rapamycin treated PfSKP1-HA or PfRBX1-HA parasites (Fig. S3). Together these data suggest that loss of PfRBX1 and PfSKP1 function results in a breakdown in membrane organisation and nuclear architecture independent of histone protein modification of DNA.

To understand the effect of loss of PfRBX and PfSKP1 function in detail the parasites lacking normal PfRBX1-HA and PfSKP1-HA expression were analysed using transmission electron microscopy (Fig. 2h). The parental strain showed no defects in either DNA segregation or merozoite formation. An intermediate defect was observed for PfRBX1 depleted parasites with many showing apparent normal DNA segregation and merozoite morphology, however, some displayed an aberrant nuclear size, incomplete partitioning of organelles into cells, and disrupted division. In contrast, *P. falciparum* parasites lacking PfSKP1 function showed severe defects in formation of new merozoites within the schizont at a stage during which one would expect normal segregation (Fig. 2h). Nuclear division appeared to be asymmetrical and asynchronous, where some merozoites contained no nuclei, whereas other merozoite-like compartments appeared to contain multiple nuclei or enlarged, undivided nuclei. In addition, nuclei were elongated with abnormal morphologies including multiple pinched protrusions. PfSKP1 conditional deleted parasites also displayed defects in rhoptry biogenesis, where the organelles were either incorrectly positioned or were electron sparse compared to the electron dense rhoptries seen in the DiCre controls. The IMC ultrastructure was also disrupted in *pfskp1* deleted parasites, where the continuity of the membranes was compromised. Collectively, these results implicate the increased importance of PfSKP1 on asexual stage parasite growth compared to PfRBX1 and the dependence of PfSKP1 to maintain normal nuclear division and segregation and viable merozoite production.

### *P. falciparum* has multiple Cullin-RING-Ligase complexes

**Fig. 3:**
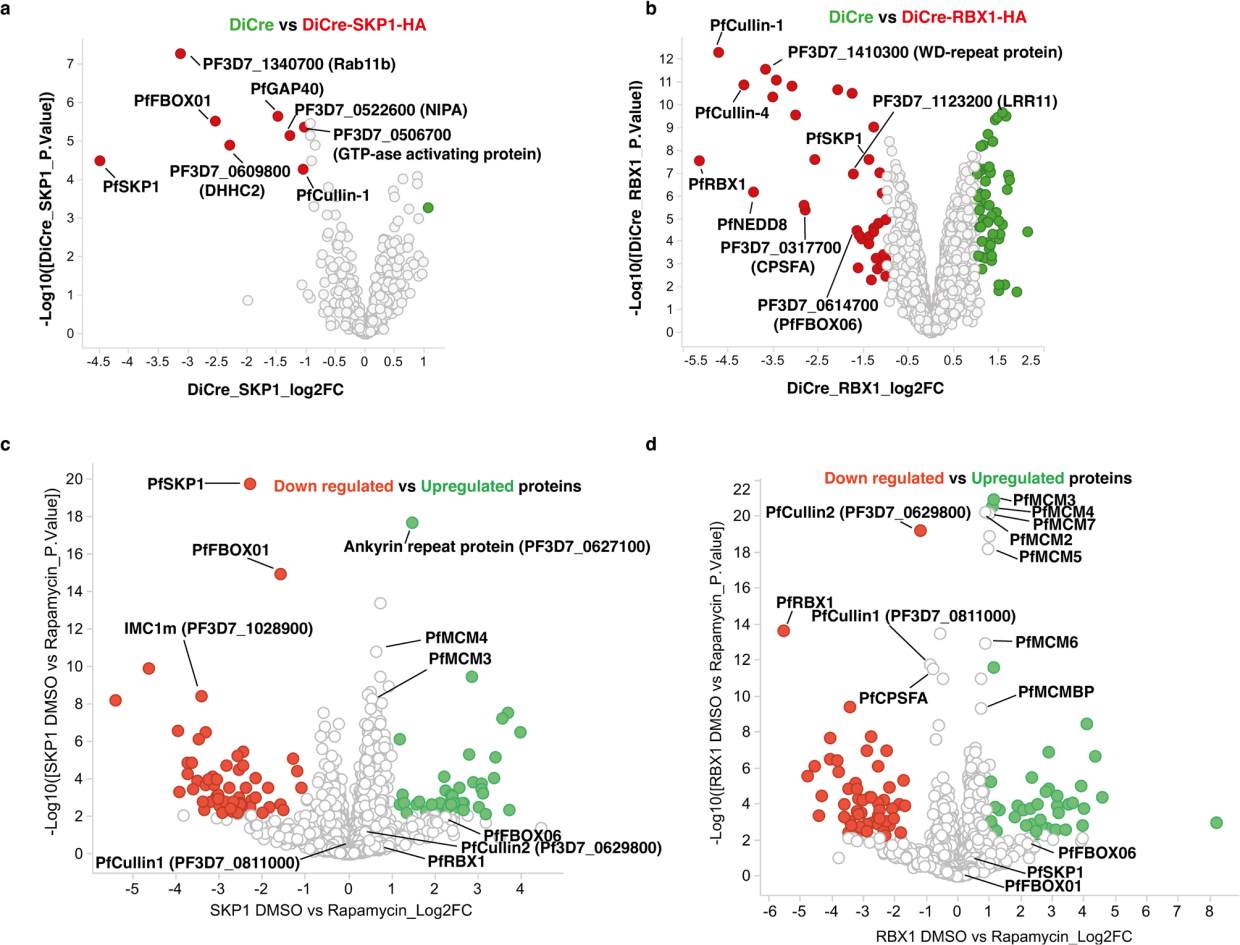
Identification of PfSKP1 and PfRBX1 interactome. (**a**) Volcano plot illustrating the log_2_ protein ratios in anti-HA immunoprecipitations from parasite lysate comparing DiCre (green) with DiCre-PfSKP1-HA (red). Proteins were deemed differentially regulated if the log2 fold change in protein expression was ≥ 1-fold and exhibited an adjusted *p* ≤ 0.05. Proteins of interest to this study are further labelled with PlasmoDB accession number. (**b**) Volcano plot illustrating the log_2_ protein ratios in anti-HA immunoprecipitations from parasite lysate comparing DiCre (green) with DiCre-PfRBX1-HA (red). Proteins were deemed differentially regulated if the log2 fold change in protein expression was ≥ 1-fold and exhibited an adjusted *p* ≤ 0.05. Proteins of interest to this study are further labelled with PlasmoDB accession number. (**c**) Volcano plot illustrating the log_2_ protein ratios in anti-HA immunoprecipitations from schizont stage parasite lysate comparing DMSO (green) with Rapamycin (red). Proteins were deemed differentially regulated if the log2 fold change in protein expression was ≥ 1-fold and exhibited an adjusted *p* ≤ 0.05. Up and down regulated proteins of specific interest to this study are highlighted in green and red respectively. (**d**) Volcano plot illustrating the log_2_ protein ratios in anti-HA immunoprecipitations from schizont stage parasite lysate comparing DMSO (green) with Rapamycin (red). Proteins were deemed differentially regulated if the log2 fold change in protein expression was ≥ 1-fold and exhibited an adjusted *p* ≤ 0.05. Up and down regulated proteins of specific interest to this study are highlighted in green and red respectively.

To understand the function of PfRBX1 and PfSKP1, potential interacting proteins were identified by co-immunoprecipitation followed by LC-MS/MS analysis. PfSKP1-HA and PfRBX1-HA tagged protein were immunoprecipitated from schizont stages of the respective transgenic parasites. In both cases, schizont stage parasites from the parental DiCre strain were used as control. The PfSKP1 pull downs revealed two groups of proteins that potentially interact (Fig. 3a). Firstly, PfSKP1 co-precipitated with both the PfCullin1 homolog PF3D7_0811100 and PfFBOX01 protein PF3D7_0619700 consistent with a PfCRL1 Skip-Cullin-FBOX complex (PfSCF). Several other proteins were also identified, that are located within the inner membrane complex in merozoites that specifically immunoprecipitated with PfSKP1. These included PfGAP40 (PF3D7_0515700), the inner membrane complex protein (PF3D7_0522600) and the palmitoytransferase PfDHHC2 (PF3D7_0609800)^40–42^. PfSKP1 also interacts with the apicomplexan specific GTP-ase Rab11b and PF3D7_0506700, a GTP-ase activating protein. Rab11b is involved in biogenesis of the inner membrane complex in *Toxoplasma gondii*^43^. These results suggested that a PfSKP1-containing SCF complex was associated with components of the IMC.

Immunoprecipitation of PfRBX1-HA identified multiple PfRBX1-containing E3 Cullin-RING Ligase (CRL) ubiquitination components of *P. falciparum* (Fig. 3b). PfRBX1-specific pulldown of PfCullin1, PfSKP1, PfNEDD8 and the PfFBOX06 protein PF3D7_0614700 indicated they were components of a PfSCF complex. PfRBX1 pulldown did not identify the PfFBOX01 protein that was isolated in the PfSKP1 interactome, suggesting that there are distinct FBOX containing PfCRL complexes in *P. falciparum*. PfRBX1 also pulled down PfCullin2 PF3D7_0629800 as well as a second putative adaptor protein CPSF-A (PF3D7_0317700). Two distinct putative substrate binding receptors, the WD-repeat protein PF3D7_1410300 and the leucine rich repeat protein PF3D7_1123300 were also identified in the PfRBX1 pulldown. These results predict the existence of multiple distinct PfRBX1 based CRL complexes that use either PfCullin1 or PfCullin2 as a scaffold and multiple adaptor-substrate receptor pairs.

To determine if the loss of PfRBX1 and PfSKP1 function influences the level of abundance of other proteins in the proteome of *P. falciparum* parasites, ring forms of the relevant transgenic parasites were treated with rapamycin to induce conditional knockout of each protein and late schizont stages analysed by LC-MS/MS. DMSO or rapamycin treated schizont stage DiCre parasite proteomes were analysed as controls for non-specific changes. No significant alterations in any proteins were observed in the DiCre control parasites due to rapamycin addition (Fig. S4). PfSKP1 deletion led to decreased levels of PfFBOX01 (PF3D7_0619700) however, there were no changes observed for PfRBX1, PfCullin1, PfCullin 2 or the PfFBOX06 protein (Fig. 3c). PfSKP1 knockdown also resulted in reduction of a protein involved in the structure of the IMC, IMC1m (Pf3D7_1028900)^44,45^. Proteins upregulated when PfSKP1 was knocked down were also identified as they could be putative substrates ubiquitinated by a PfSKP1-containing E3 ligase complex and consequently degraded by the proteosome. Increased levels of Ankyrin repeat protein PF3D7_0627100 and the minichromosome maintenance (MCM) proteins PfMCM3 (PF3D7_0527000) and PfMCM4 (PF3D7_1317100) were detected. The MCM complex is essential for correct DNA replication in eukaryotic cells^46^. Currently there is no known function on the ankyrin repeat protein in *P.falciparum*, but it is known that a conserved ankyrin repeat containing protein regulates parasite invasion in *T. gondii*^47^.

Upon PfRBX1 knock down decreases in the protein levels of both PfCullin 1 and 2 and the putative adaptor protein PfCPSF-A were detected (Fig. 3d). No changes were observed in the levels of PfSKP1 or the identified PfFBOX proteins. Increases in the expression levels of all six MCM complex proteins PfMCM2-MCM7 as well as its accessory protein PfMCMBP were also identified suggesting abundance or stability of this complex increased in the absence of PfRBX1 function. Collectively, these results suggest that *P. falciparum* blood stages have multiple E3 ligases; a PfSCF complex utilising PfFBOX01 with PfCullin1 and diverse PfCullin1 and PfCullin2 based PfCRL complexes that use distinct substrate adaptors and receptors.

### An F-box protein linked to a SCF complex is essential for survival of *P. falciparum*

**Fig. 4:**
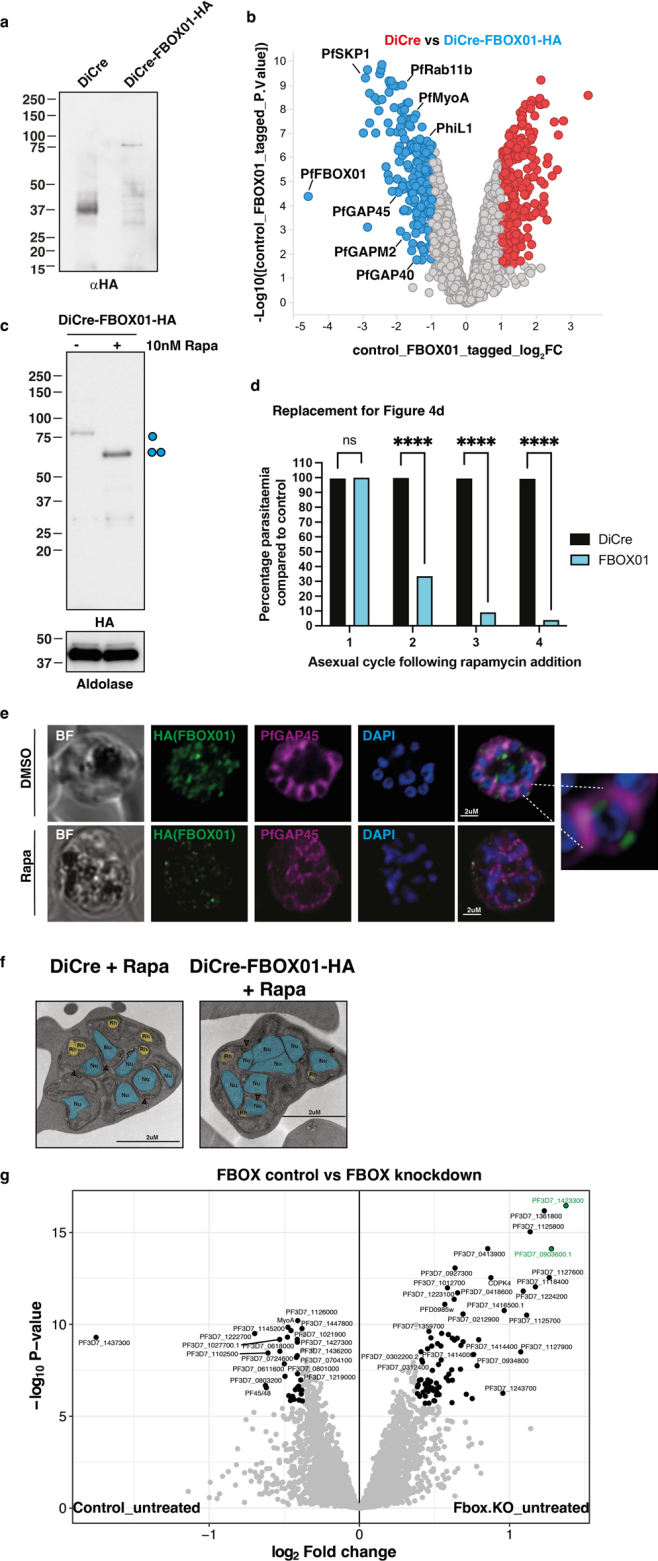
Identification of PfFBOX01 substrate receptor. (**a**) Immunoblots showing C-terminal tagging of DiCre-PfFBOX01 transgenic strain parasites by anti-HA antibodies. (**b**) Volcano plot illustrating the log_2_ protein ratios in anti-HA immunoprecipitations from parasite lysate comparing DiCre (red) and DiCre-PfFBOX01-HA (blue). Proteins were deemed differentially regulated if the log2 fold change in protein expression was ≥ 1-fold and exhibited an adjusted *p* ≤ 0.05. Proteins of interest to this study are further labelled with PlasmoDB accession number. (**c**) Immunoblots of schizont stage DiCre-PfFBOX01-HA parasites treated with 0 or 10nM rapamycin for 48 h. DiCre-Rapamycin mediated excision and protein loading assessed by anti-HA and anti-Aldolase antibodies respectively. Full-length and truncated PfRBX1-HA is denoted by one and two cyan circles respectively. (**d**) Growth curve of ring stage DiCre and DiCre-PfFBOX01-HA parasites grown on DMSO or 10nM Rapamycin for 1–3 intraerythrocytic cycles. Parasitemia is assessed as a percentage compared to growth of control parasite grown on DMSO. Data are mean ± s.e.m for *n* = 3 biological replicates with 2 technical duplicates and compared by two-way Anova (Dunnett’s multiple comparison test. (**e**) Immunofluorescence analysis of DMSO and Rapamycin treated DiCre-PfFBOX01-HA schizont stage parasites. FBOX01 levels, parasite membrane integrity and DNA levels were visualised using anti-HA, anti-PfGAP45 antibodies and DAPI staining respectively. Inset shows magnification of merozoite within the schizont stage parasite. (**f**) Transmission electron microscopy of Rapamycin treated DiCre-PfFBOX01-HA schizont stage parasites. The nuclei and rhoptries have been manually annotated and coloured blue and yellow respectively using a trace feature from Adobe Illustrator that identifies these organelles. (**g**) Volcano plot illustrating the log_2_ protein ratios in anti-HA immunoprecipitations from schizont stage parasite lysate comparing DMSO and Rapamycin treated DiCre-PfFBOX01-HA. Proteins were deemed differentially regulated if the log2 fold change in protein expression was ≥ 1-fold and exhibited an adjusted *p* ≤ 0.05. Up regulated proteins of specific interest to this study are highlighted in green.

The role of the PfFBOX01 protein in the PfSCF complex was analysed by creating a transgenic parasite where the full-length protein would be C-terminally HA-tagged and upon rapamycin treatment, the Floxed FBOX domain of the protein excised and a 3xHA-NeonGreen tag appended to the C-terminus resulting in a truncated protein. The HA tagging of the full-length PfFBOX01 protein was confirmed by immunoblot (Fig. 4a, Fig. S10). Anti-HA beads were used for immunoprecipitation of PfFBOX01-HA from transgenic schizont stage parasites were analysed by LC-MS/MS and confirmed this protein interacted with PfSKP1 (Fig. 4b). In addition, several rhoptry, glideasome, inner membrane complex proteins and the GTPA-ase subunit Rab11b were significantly enriched in the PfFBOX01 pulldown.

Western blot analysis of rapamycin vs. DMSO treated PfFBOX01-HA schizont stage parasites showed complete excision of the full-length HA tagged protein and the appearance of the FBOX domain-less HA-NeonGreen protein (Fig. 4c, Fig. S10). Analysis of PfFBOX01-HA parasites across multiple growth cycles showed a rapid decrease in parasitemia upon rapamycin treatment, indicating that PfFBOX01 function was essential for survival of intra-erythrocytic *P. falciparum* (Fig. 4d). PfFBOX01 expression peaks at schizogony, and consequently we analysed PfFBOX01-HA schizonts treated with DMSO or rapamycin by immunofluorescence analysis (Fig. 4e). In the absence of rapamycin, full-length PfFBOX01 protein localised at the periphery of the developing merozoite. The almost complete knockdown of the PfBOX01 protein expression caused dysregulation of both nuclear division and membrane organisation of schizonts, a phenotype like that of parasites lacking PfSKP1 function. Expression of truncated PfFBOX01 protein was restricted to puncta within the parasite cytoplasm and distinctly away from the apical and basal poles. Electron microscopy of rapamycin-treated PfFBOX01-HA schizonts showed that these parasites had a morphology like that seen for those lacking PfSKP1 function wherein aberrant nuclear division, abnormal merozoite segmentation and the absence of electron-dense rhoptries was observed (Fig. 4f). These results suggest that PfFBOX01 functions with PfSKP1, potentially in concert as a complex to regulate viable schizont production.

To determine if lack of PfFBOX01 function influenced the level of other proteins DMSO and rapamycin treated PfFBOX01-HA schizonts were analysed by LC-MS/MS (Fig. 4g). There were no significant changes in the level of proteins within the MCM complex, suggesting that PfFBOX01 was not directly responsible for any change in protein levels of PfMCM4 or other subunits. However, there was an increase in abundance of several essential *P. falciparum* proteins, upon rapamycin treatment, including protein phosphatase 7 PF3D7_1423300, CRAL/TRIO domain containing protein PF3D7_1127600 and the uncharacterised protein PF3D7_0903600.1. Of these proteins, PF3D7_0903600 was also identified as a specific PfFBOX01 interactor by anti-HA immunoprecipitation (Fig. 4g).

In human cells, proteins upregulated in the cell upon FBOX deletion tend to be substrates of the associated E3 ligase complex^48^. To validate the changes in abundance of proteins following knockdown of PfFBOX01-HA the proteins PF3D7_1423300 and PF3D7_0903600.1 were tagged at the C-terminus with FLAG epitopes (Fig. S5 and S13). Anti-FLAG immunoblots of schizont-stage parasites treated with DMSO or rapamycin showed that while almost complete depletion of PfFBOX01 was observed, no significant changes in the levels of the two putative substrate proteins were detected (Fig. S5 and S13). However, immunoprecipitation of PfFBOX01-HA did identify a specific interaction with PF3D7_0903600.1 but not with PF3D7_1423300 (Fig. S5 and S13). Collectively, these results suggest that while PfFBOX01 was present as a PfCullin1-PfSKP1 based complex and the PfFBOX01 protein is essential to produce schizont stage parasites, this E3 ligase complex may not be mediating ubiquitination-dependent degradation of target substrates at this lifecycle stage.

### RBX1-SKP1 deletion leads to dysregulation of a DNA-replication complex

**Fig. 5:**
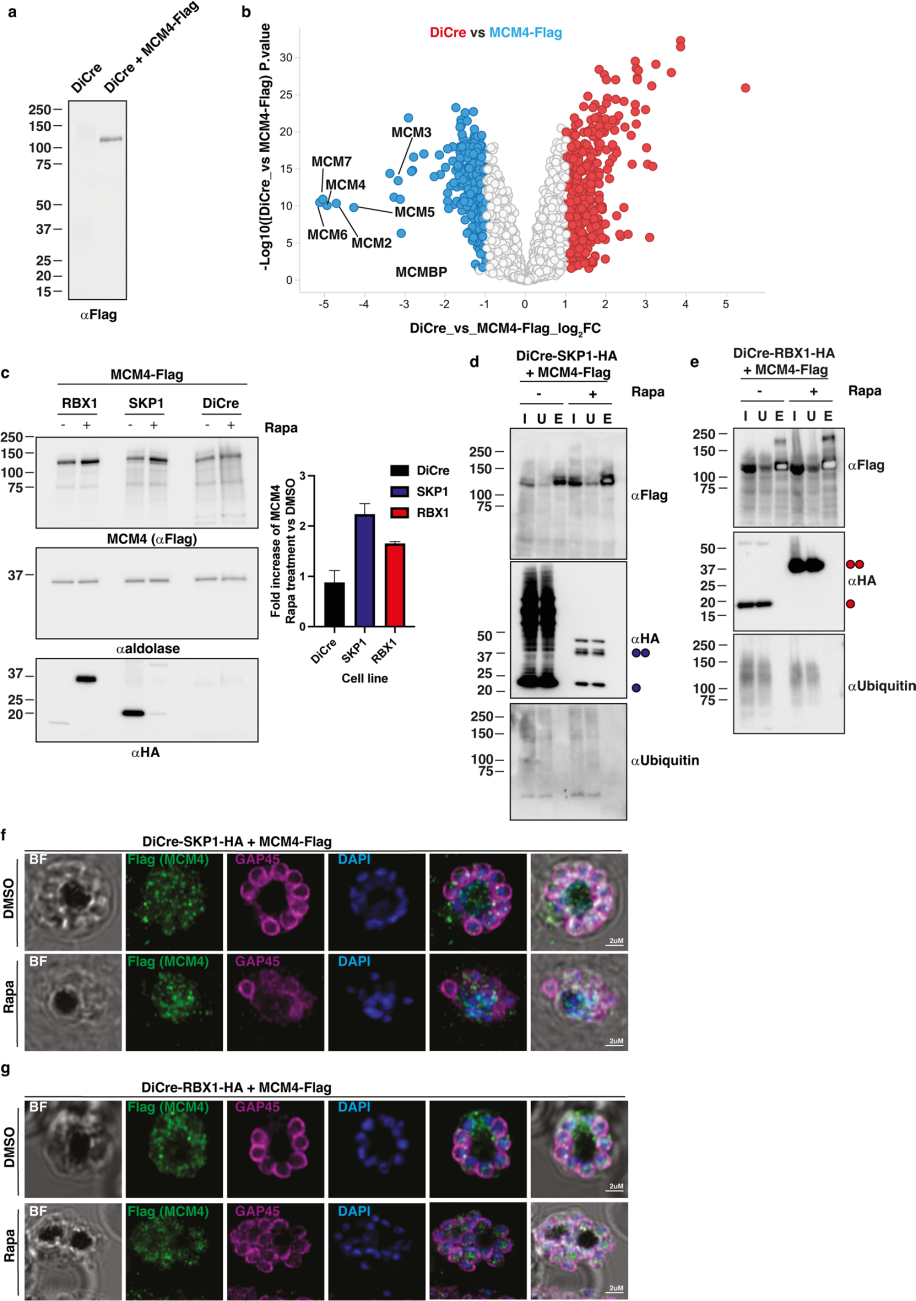
Identification of MCM complex in *P. falciparum*. (**a**) Immunoblots showing C-terminal tagging of PfMCM4 in DiCre transgenic strain parasites by anti-Flag antibodies. (**b**) Volcano plot illustrating the log_2_ protein ratios in anti-FLAG immunoprecipitations from parasite lysate comparing DiCre (red) and PfMCM4-Flag (blue). Proteins were deemed differentially regulated if the log2 fold change in protein expression was ≥ 1-fold and exhibited an adjusted *p* ≤ 0.05. Proteins of interest to this study are further labelled with protein names. (**c**) Immunoblot of MCM4-Flag containing DiCre, PfSKP1-HA and PfRBX1-HA schizont stage parasites treated with DMSO or rapamycin. Anti-Flag, Aldolase and HA antibodies were used to identify levels of PfMCM4, loading and PfSKP1/PfRBX1 protein levels respectively. Densitometry of *n* = 2 western blots with mean ± s.d are plotted next to immunoblot. (**d**) Immunoblot of anti-Flag immunoprecipitation of MCM4-Flag from DiCre-PfSKP1-HA + MCM4-Flag transgenic schizont stage parasites pre-treated with DMSO or 10 nM Rapamycin. Input (I), Unbound (U) and Eluate (E) fractions were analysed using antibodies against MCM4 (anti-Flag), Ubiquitin (anti-ubiquitin) and PfSKP1 (anti-HA). Full-length and truncated PfSKP1-HA is denoted by one and two blue circles respectively. Note that the anti-HA panel has been overexposed so that the SKP1 HA-Neon Green fusion can be observed. (**e**) Immunoblot of anti-Flag immunoprecipitation of MCM4-Flag from DiCre-PfRBX1-HA + MCM4-Flag transgenic schizont stage parasites pre-treated with DMSO or 10nM Rapamycin. Input (I), Unbound (U) and Eluate (E) fractions were analysed using antibodies against MCM4 (anti-Flag), Ubiquitin (anti-ubiquitin) and PfRBX1 (anti-HA). Full-length and truncated PfRBX1-HA is denoted by one and two red circles respectively. (**f**) Immunofluorescence analysis of MCM4-Flag tagged DiCre-PfSKP1-HA strain schizont stage parasites. Parasites were treated with DMSO or Rapamycin to induce PfSKP1 deletion and PfMCM4, parasite membrane integrity and DNA levels were visualized with anti-Flag, anti-PfGAP45 antibodies and DAPI staining respectively. (**g**) Immunofluorescence analysis of MCM4-Flag tagged DiCre-PfRBX1-HA strain schizont stage parasites. Parasites were treated with DMSO or Rapamycin to induce PfRBX1 deletion and PfMCM4, parasite membrane integrity and DNA levels were visualized with anti-Flag, anti-PfGAP45 and DAPI respectively.

To determine if PfCRL complexes played a direct role in regulation of the PfMCM complex immunoprecipitations were performed and analysed by LC-MS/MS. Immunoprecipitation of PfMCM4 tagged with a FLAG epitope (Fig. 5a, Fig. S11) significantly enriched all remaining components of the predicted PfMCM complex PfMCM2/3/5/6/7 as well as the previously characterised PfMCM binding protein (PfMCMBP)^49^ (Fig. 5b). There was no evidence for the presence of MCM8, 9 accessory proteins in the *P. falciparum* MCM complex whereas these are present in other eukaryotes^50,51^. These results identified an MCM complex expressed in the asexual blood stages of *P. falciparum*.

To understand impacts on the PfMCM complex upon the loss of PfRBX1 and PfSKP1 function, we introduced a C-terminal FLAG-tag on PfMCM4 in both PfRBX1-HA and PfSKP1-HA conditional knockout strains. Rapamycin treatment to induce deletion of the E3 ligase components followed by western blot analysis showed increased abundance of PfMCM4, thus validating the whole cell proteomic data (Fig. 5c and Fig. S11). Immunoprecipitation of the same parasite material revealed that while higher levels of PfMCM4 were induced by rapamycin treatment for both parasite strains, the isolated protein was not ubiquitinated at altered levels compared to controls, and there was no evidence for co-pulldown of PfSKP1 or PfRBX1 (Fig. 5d, e and Fig. S11).

Rapamycin-mediated deletion of PfRBX1 or PfSKP1 did not lead to a change in the localisation of PfMCM4, suggesting that the increased protein levels was not due to mislocalisation. PfMCM4 was shown to be localised close to the nucleus of schizont stage parasites (Fig. 5f, g). To determine if the composition of the PfMCM complex was altered upon PfRBX1 or PfSKP1 deletion schizont stage parasites were treated with DMSO or rapamycin and PfMCM4-FLAG protein immunoprecipitated and analysed by LC-MS/MS. There were no significant changes in the composition of the PfMCM4 interactome upon deletion of the PfSKP1 (Fig. S6). There was, however, a significant decrease in the level of PfCullin 1 and PfCullin2 binding by PfMCM4 upon deletion of PfRBX1 suggesting that the CRL complexes mediated by PfRBX1 physically interact with the PfMCM complex (Fig. S6). In other eukaryotes, MCM protein binding to DNA is dynamically controlled by a cycle of phosphorylation-dephosphorylation of the individual complex components (reviewed in^52^). Therefore, we analysed the immunoprecipitated material for any changes in the abundance in MCM complex phosphopeptides however, no biologically significant changes were identified (Fig. S7). Collectively these results suggested that while there was an increase of PfMCM4 abundance in parasites upon the knockdown of PfRBX1 or PfSKP1 there was no change in PfMCM4 localisation, PfMCM complex composition or phosphorylation-dephosphorylation status of the individual components. This suggests a ubiquitination independent mechanism whereby PfRBX1 and/or PfSKP1 regulates PfMCM4 levels in *P. falciparum* schizonts.

### Identification of a novel CRL4-based substrate adaptor PfCPSFA

In human cells, the substrate adaptor proteins DDB1 interacts with a Cullin4A- based E3 ligase complex and targets substrate proteins for ubiquitination in response to DNA damage^53,54^. These substrates include the replication licensing factor CDT1, which assists in loading the MCM complex onto DNA^53–55^. PfCPSFA, a PfRBX1 interacting protein that was downregulated upon knockdown of PfRBX1 contains ~ 20% sequence identity with human DDB1. Therefore, we investigated whether PfCPSFA was required for either the homeostasis of the MCM complex or in schizont development.

**Fig. 6:**
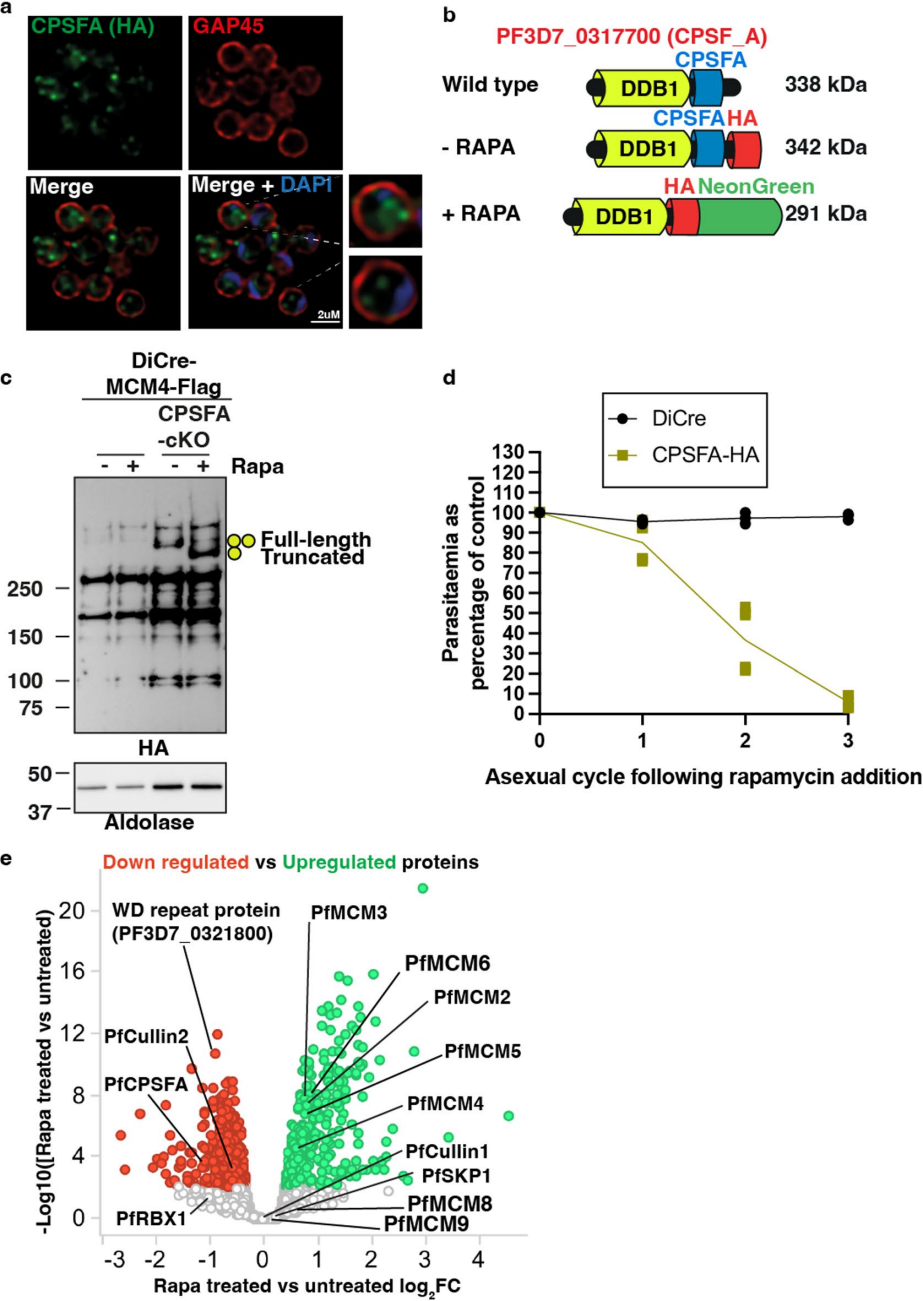
Characterisation of PfCPSFA-HA and role in PfMCM4 levels. (**a**) Immunofluorescence analysis of DMSO treated DiCre-PfCPSFA-HA schizont stage parasites. PfCPSFA levels, parasite membrane integrity and DNA levels were visualised using anti-HA, anti-PfGAP45 antibodies and DAPI staining respectively. Inset shows magnification of merozoite within the schizont stage parasite. (**b**) Schematic of protein size of wild type, HA-tagged and truncated PfCPSFA in DiCre-PfCPSFA-HA transgenic parasite strain. CPSFA domain in blue, HA tag in red and Neon Green domain in green colours respectively. (**c**) Immunoblot of MCM4-Flag containing DiCre and PfCPSFA-HA schizont stage parasites treated with DMSO or rapamycin. Anti-Flag, Aldolase and HA antibodies were used to identify levels of PfMCM4, loading and PfSKP1/PfRBX1 protein levels respectively. Full-length and truncated PfCPSFA-HA is denoted by one and two green circles respectively. (**d**) Growth curve of ring stage DiCre and DiCre-PfCPSFA-HA parasites grown on DMSO or 10nM Rapamycin for 1–3 intraerythrocytic cycles. Data are mean ± s.e.m for *n* = 3 biological replicates with 2 technical duplicates and compared by two-way Anova (Dunnett’s multiple comparison test. Parasitemia is assessed as a percentage compared to growth of control parasite grown on DMSO. (**e**) Volcano plot illustrating the log_2_ protein ratios in anti-HA immunoprecipitations from schizont stage parasite lysate comparing DMSO (red) and Rapamycin treated DiCre-PfCPSFA-HA (green). Proteins were deemed differentially regulated if they exhibited an adjusted *p* ≤ 0.05. Up and down regulated proteins of specific interest to this study are highlighted in green and red respectively.

A PfDiCre-based conditional knockout strain (PfCPSFA-HA) was constructed and in the absence of rapamycin, PfCPSFA was localised to punctate structures within the parasite cytoplasm, like the localisation observed for PfRBX1 (Fig. 6a). This transfected *P. falciparum* strain also expressed a FLAG epitope tagged PfMCM4 protein, and addition of rapamycin activated the deleted of the CPSFA domain which transposed a HA-NeonGreen tag in frame with the truncated N-terminal protein (Fig. 6b). Near complete deletion of full-length PfCPSFA was observed in the first cycle of rapamycin addition, with the associated appearance of the HA-NeonGreen tagged truncated protein (Fig. 6c and Fig. S12). Ring-stage parasites were treated with rapamycin and growth monitored across three asexual life cycles (Fig. 6d). PfCPSFA-HA parasitemia was unaffected in the cycle following rapamycin addition but significantly decreased in the 2nd and 3rd cycle suggesting PfCPSFA was essential for parasite survival and that it had a phenotype like that observed in the absence of function. PfCPSFA dependent proteomic changes were analysed for rapamycin-treated PfCPSFA schizonts by LC-MS/MS (Fig. 6e). Addition of rapamycin led to the expected de-stabilisation of PfCPSFA. There was significant decrease in PfCullin2 abundance but not of the PfCullin1 variant, suggesting that PfCPSFA functions via a PfCullin2-based complex. The downregulation of a single WD-repeat protein (PF3D7_0321800), out of 29 WD-repeat proteins detected during the proteomic analysis, was indicative of a substrate receptor that also functions through the PfCPSFA based E3 ligase complex. PfCPSFA deletion also led to a decrease in the level of PfFBOX06, suggesting that PfFBOX06 may also utilise PfCPSFA as a substrate adaptor. We also identified proteins that increased in abundance upon PfCPSFA deletion. Of note was the increased abundance of 5 of the 6 core PfMCM complex subunits upon PfCPSFA deletion. Collectively these data suggest the increased abundance of the MCM complex seen after PfRBX1 deletion likely occurs through the PfCPSFA adaptor protein influencing these proteins’ stability.

## Discussion

Due to their roles in governing critical processes in cellular homeostasis, CRL complexes are attractive drug targets. An understanding of human CRL complexes, including organisational information of the complexes as well as identification of their specific substrates, has allowed structure-based rational design of CRL inhibitors (reviewed in^56^). These inhibitors can arrest the function of various human E3 ligases including CRL1 and CRL4 based complexes^57–61^. In the absence of sequence-identical homologues, several inhibitors of human E3 ligases have also shown in vitro efficacy against *P. falciparum* growth suggesting the existence of structurally homologous E3 ligases in malaria parasites that infect humans^62^. To address the lack of biochemical and functional information regarding *P. falciparum* E3 ubiquitin ligases, our study assessed the function of ubiquitination and PfCRL complexes in the asexual parasite blood stage of *P. falciparum*.

*P. falciparum* protein ubiquitination increases through the ring, trophozoite and schizont intra-erythrocytic stages to reach a maximum within merozoites^15^. There is evidence for ubiquitination of many proteins in *P. falciparum* life cycle stages, with overlap for some at all stages of the asexual life cycle. The E1 inhibitor MLN7243 as well as the conditional knockout of the putative *P. falciparum* E1 enzyme target PF3D7_1333200 allowed ring stage parasite to mature until early schizont, at which point they arrested in growth strongly suggesting an increased dependence on activated ubiquitin for merozoite development^15^. Activation of the CRL complexes in parasites by neddylation also appears essential as the Nedd8 inhibitor MLN4924 kills *Plasmodium* parasites, while HA-tagged PfNEDD8 protein also interacts with both PfCullin isoforms^33^. Conditional knockout of all components of the PfCRLs identified by our study also arrest parasite development at schizogony. It is interesting that even though there is evidence for the ubiquitination of essential proteins in the preceding stages^15^the absence of key ubiquitin mediators appears to be especially required during schizogony.

In a canonical PfCRL complex containing PfCullin1, the only significant substrate receptor identified as a PfSKP1 interactor appears to be PfFBOX01. Compared to human cells that contain up to 70 variants^63^only two FBOX01 receptors were predicted within the *P. falciparum* genome; PF3D7_0619700 (PfFBOX01) and PF3D7_0614700 (PfFBOX06). While PfFBOX06 was expressed in the asexual blood stages and appears to interact with PfRBX1, only PfFBOX01 was isolated as a PfSKP1 binder by both immunoprecipitation and whole cell proteomics following conditional knockdown. PfFBOX06 may therefore function as part of a PfRBX1-containing CRL complex that utilises an alternative substrate adaptor. The paucity of *Plasmodium* FBOX proteins is intriguing and suggests presence of stable CRL complexes rather than the dynamic forms seen in higher eukaryotes. In these species the protein CAND1 is essential for the switching of FBOX proteins on a single SCF complex and the disassembly of CRL complexes^11,64^however there is no direct homologue for a PfCAND1 protein, and our immunoprecipitation and knockdown data have failed to identify a suitable alternative candidate. Given the presence of only few PfFBOX proteins and a lack of a known dissociation factor, PfCRL complexes may not undergo dynamic switching of substrate receptors and could exist simultaneously as independent stable structures. These data suggest a non-degradative role in parasite development for PfFBOX01 and the PfSCF complex within the asexual schizont. In agreement with our findings, recent data from the murine *P. berghei* parasite suggest that the SCF^FBOX01^ complex may have important degradative roles in the sexual stages of the life cycle^65^. As this study was limited to analysis of asexual stage *P. falciparum* infection, future work will be undertaken to investigate the role of all PfCRL complexes in sexual gametocyte stages.

In schizonts PfFBOX01 and PfSKP1 both interact specifically with proteins that form the inner membrane complex. Indeed, the FBOX01 protein from the related apicomplexan parasite *Toxoplasma gondii* localises to the daughter cell scaffold, from which the IMC arises^66^. While the process of inner membrane complex formation during schizogony is relatively well understood, the mechanism of its disassembly, a process completed within 60 min of successful parasite invasion, remains unknown^67,68^. Several observations suggest that ubiquitination regulates organisation and disassembly of the IMC: the association of a PfSCF complex with inner membrane complex components, the essentiality of this complex for inner membrane complex organisation, and the comparable expression profiles of PfFBOX01 and known IMC proteins wherein a peak in late schizogony is followed by a rapid decrease upon invasion. Based on these data we propose a model in which the PfFBOX01-containing PfSCF complex binds to and stabilises the IMC during schizogony and then degrades it in a ubiquitin-dependent manner immediately following invasion of merozoites.

In addition to PfSKP1, PfRBX1 binds only one other protein that has homology to a known substrate adaptor. Given the homology between human DDB1 and PfCPSFA, the Cullin subunit binding this complex PF3D7_0629800 is likely to be of the Cullin 4 class rather than Cullin 2 as predicted. Human Cullin 4 complexes all use DDB1 as a substrate adaptor and DDB1-Cullin4 associated factors (DCAFs) as substrate receptors, of which many DCAF members contain WD-repeat domains^69^. DCAF-DDB1-CRL4 complexes are involved in varied cellular processes such as cell cycle regulation, DNA damage response and maintenance of genomic integrity^70,71^. It is of particular interest that PfCPSFA has homology to DDB1, and it appears to regulate the levels of the WD-repeat protein PF3D7_0321800. While this study was under review, Rizvi et al. also independently identified PfCPSFA and PF3D70321800 as interacting partners of PfCullin2 ^72^. However, at this stage we have not proven that the MCM complex is directly linked to PfCRL4 complex, however, our proteomic data is suggestive of an interaction. Changes in protein level of multiple MCM complex components upon PfRBX1 and PfCPSFA knockdown, as well as decreased PfCullin1 and PfCullin2 binding by PfMCM4 in the absence of PfRBX1 are consistent with a link between the PF3D7_0321800-PfCPSFA-PfCRL4 complex and DNA replication. We have shown that the increased levels of the MCM complex in PfRBX1 or PfSKP1 depleted parasites was not due to changes in the ubiquitination or phosphorylation status of PfMCM components.

**Fig. 7:**
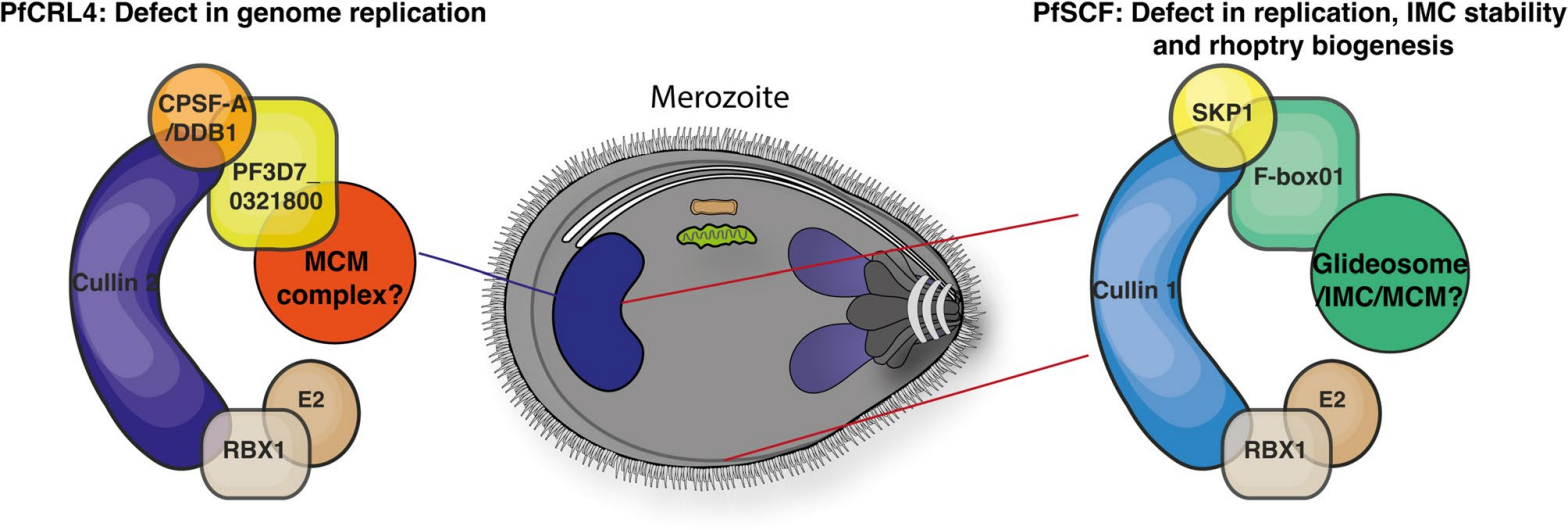
Model of PfCRL complex function in *P. falciparum* schizonts. PfSCF and PfCRL complexes identified to date function during *P. falciparum* schizogony and are essential for DNA replication, rhoptry biogenesis and normal merozoite development.

In this study we have identified the repertoire of CRL components active within the *P. falciparum* asexual blood stage. We propose the existence of at least two distinct CRLs, including a Cullin1 based PfSCF and a Cullin4 based PfCRL4 complex (Fig. 7). While components of both complexes influence normal schizogony, the PfSCF complex appears to be involved in DNA replication, IMC biogenesis and stability and rhoptry biogenesis while the PfCPSFA containing PfCRL4 complex was linked to DNA replication.

## Electronic supplementary material

Below is the link to the electronic supplementary material.

Supplementary Material 1

## Data Availability

The datasets for mass spectrometry proteomics has been deposited to the ProteomeXchange Consortium via the PRIDE 31 partner repository with the Project Name: Functional characterisation of Cullin-Ring-Ligases in Plasmodium falciparum Project accession: PXD063957.
