# Supplementary material for: Functional characterisation of components in two Plasmodium falciparum Cullin-RING-Ligase complexes
Source: Sci Rep. 2025 Jul 1;15:21359. doi: 10.1038/s41598-025-05342-0 (PMC12218279; doi:10.1038/s41598-025-05342-0)
Supplement: Supplementary file 1 — Supplementary Material 1 [file 41598_2025_5342_MOESM1_ESM.pdf]

Functional characterisation of components in two *Plasmodium falciparum* Cullin-RING-Ligase complexes

Danushka Marapana [1,2,*], Simon A. Cobbold [1,2], Michal Pasternak [1,2], Gerald J. Shami [3,4], Stuart A. Ralph [3], Sash Lopaticki [1,5], Jumana Yousef [1,2], Vineet Vaibhav [1,2], Laura F. Dagley [1,2], David Komander [1,2] and Alan F. Cowman [1,2,*]

[1] The Walter and Eliza Hall Institute of Medical Research, Parkville 3052, Australia

[2] Department of Medical Biology, The University of Melbourne, Melbourne 3010, Australia

[3] Department of Biochemistry and Pharmacology, Bio21 Molecular Science and Biotechnology Institute, The University of Melbourne, Melbourne 3010, Australia

[4] School of Medical Sciences (Molecular and Cellular Biomedicine) & Australian Centre for Microscopy and Microanalysis, The University of Sydney, NSW 2006

[5] Department of Infectious Diseases, Doherty Institute, University of Melbourne, Parkville, 3010, Australia

[*] Correspondence to: cowman@wehi.edu.au and marapana@wehi.edu.au

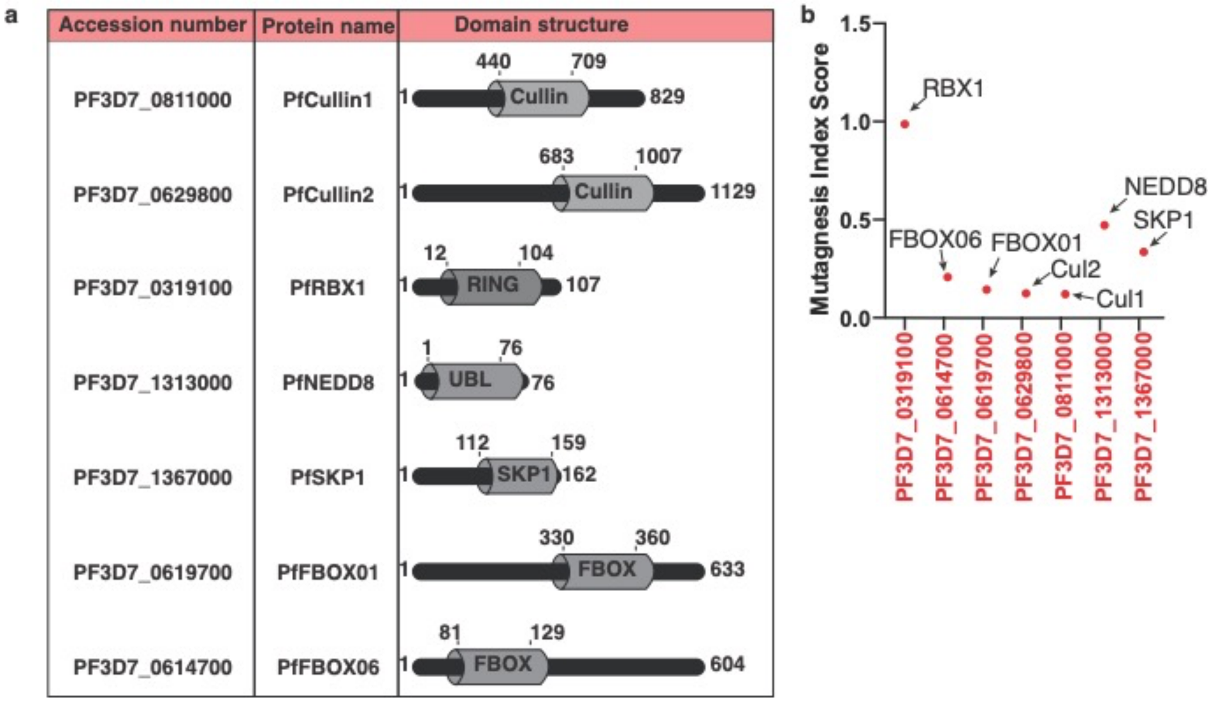

Supplementary Figure 1: Identification of key PfCRL components

S1A: PlasmoDB accession numbers, protein name and domain structure of bioinformatically identified Pf Cullin RING Ligase components. Key domains of each protein and location specifically annotated.

S1B: Bioinformatically identified PfCRL subunits plotted based on their essentiality for asexual stage growth as reported by Zhang et al 2018.

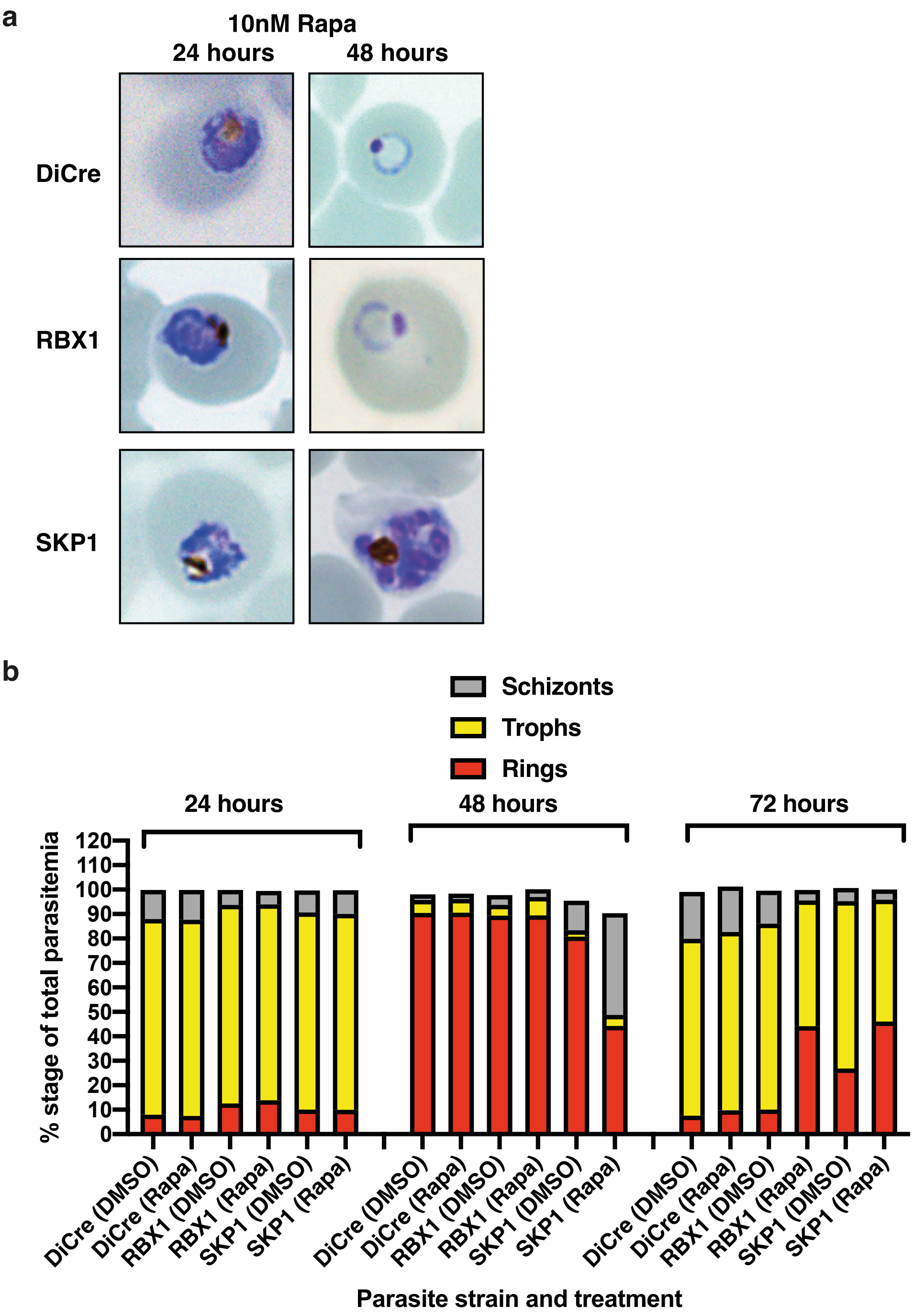

Supplementary Figure 2. Phenotype of PfRBX1-HA and PfSKP1-HA cKO parasites.

a. Giemsa-stained images of DiCre, DiCre-PfRBX1-HA and DiCre-PfSKP1-HA transgenic strain ring-stage parasites treated with 10 nM Rapamycin for 24 and 48 hours. b. Growth analysis of DiCre, DiCre-PfRBX1-HA and DiCre-PfSKP1-HA transgenic strain ring-stage parasites treated with 10 nM rapamycin for 24, 48 and 72 hours. At each time point, the percentage of ring, trophozoite and schizont stages were measured compared to DMSO treated transgenic strain control.

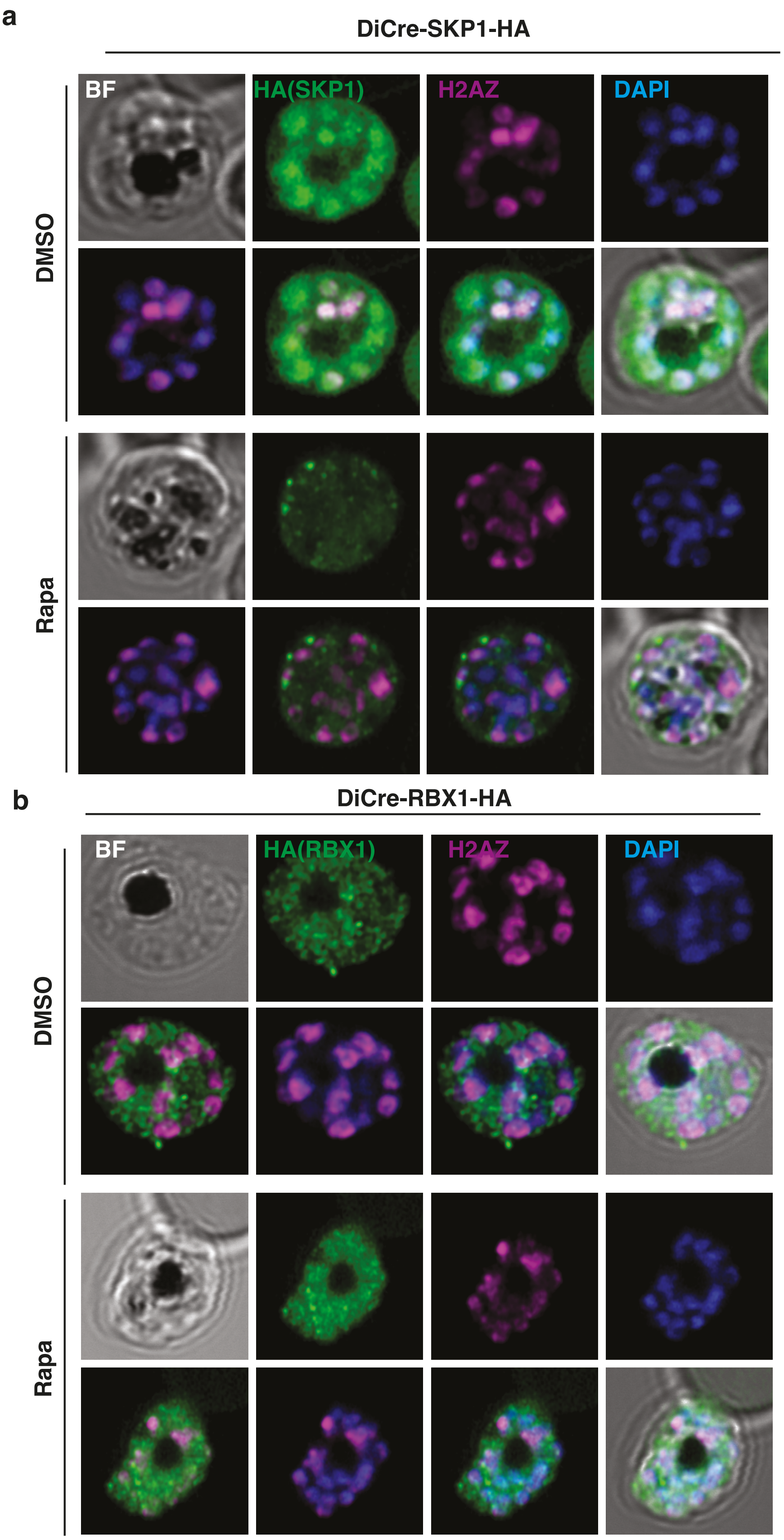

Supplementary Figure 3. H2AZ distribution in PfRBX1-HA and PfSKP1-HA cKO parasites. a. Immunofluorescence analysis of DMSO (top 2 panels) and Rapamycin treated

(bottom 2 panels) DiCre-PfSKP1-HA schizont stage parasites. PfSKP1 levels, H2AZ and DNA levels were visualised using anti-HA, anti-PfH2AZ antibodies and DAPI staining respectively. b. Immunofluorescence analysis of DMSO (top 2 panels) and Rapamycin treated (bottom 2 panels) DiCre-PfRBX1-HA schizont stage parasites. PfRBX1 levels, H2AZ and DNA levels were visualised using anti-HA, anti-PfH2AZ antibodies and DAPI staining respectively.

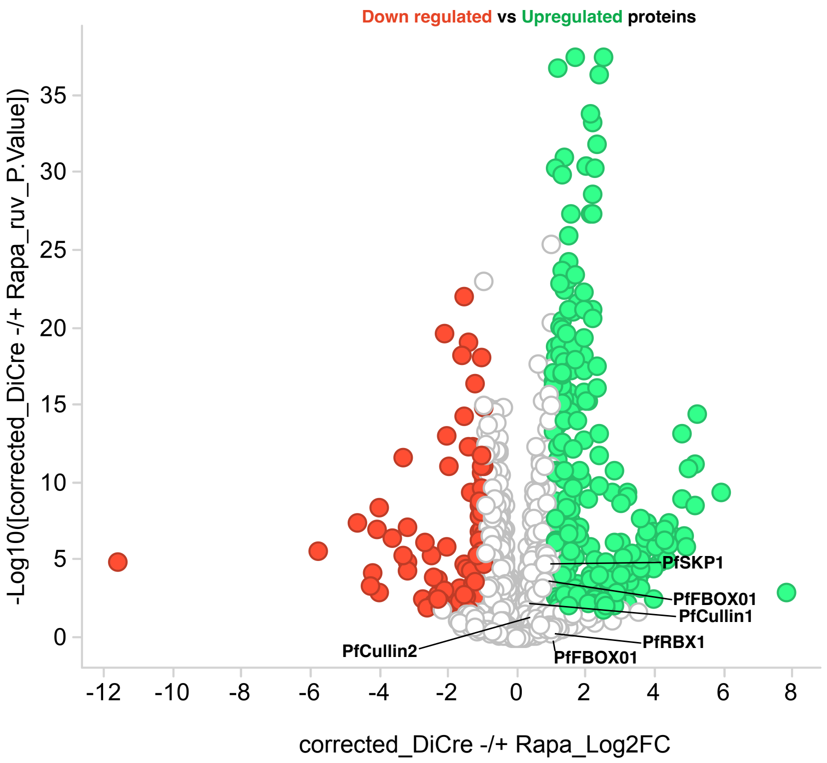

Supplementary Figure 4. Whole cell proteomic changes following rapamycin treatment of DiCre parental strain. Volcano plot of LC-MS/MS analysis of DMSO and Rapamycin treated DiCre strain schizont stage parasites. Proteins of specific interest to this study are annotated.

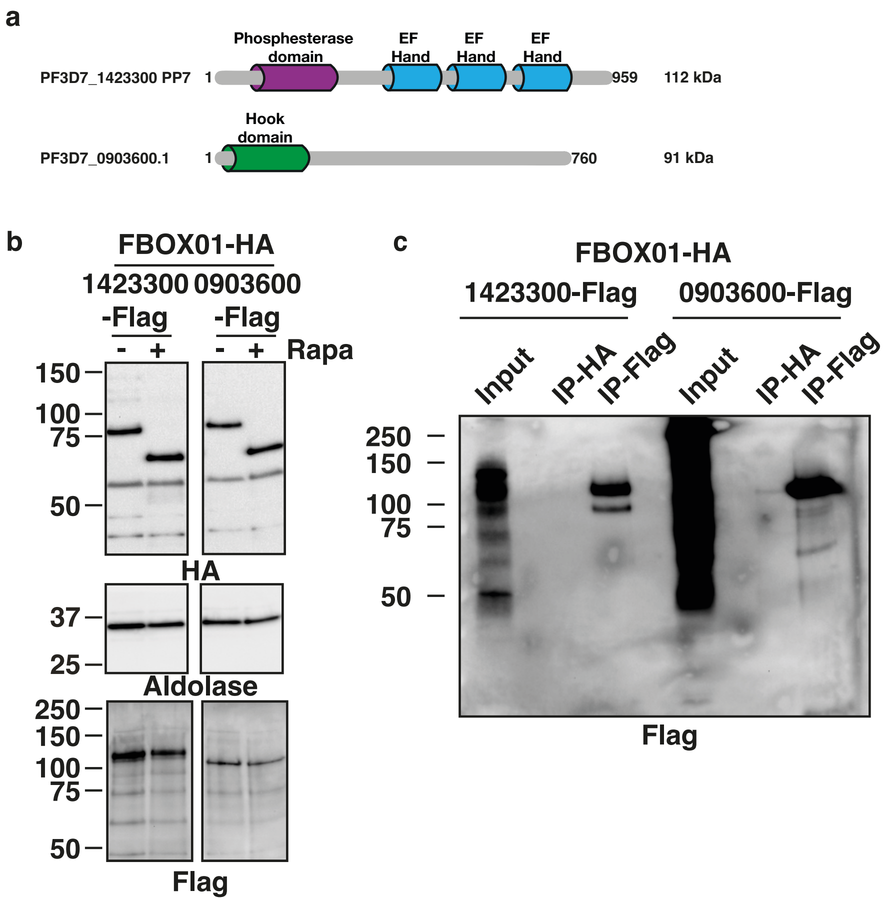

Supplementary Figure 5: Characterisation of potential PfFBOX01 interacting proteins. a. Domain map of PF3D7_1423300 and PF3D7_0903600.1 proteins and molecular weights. b. Immunoblot of PF3D7_1423300-Flag or PF3D7_0903600.1-Flag containing PfFBOX01-HA schizont stage parasites treated with DMSO or rapamycin. Anti-Flag, Aldolase and HA antibodies were used to identify levels of 1423300/0903600.1, loading and PfFBOX01 protein levels respectively. c. Immunoblot of immunoprecipitation from PfFBOX01-HA + 1423300-Flag or PfFBOX01-0903600.1-Flag tagged parasite lysate. PfFBOX01-HA or Flag-tagged PF1423300/0903600.1 proteins were isolated using anti-HA and anti-Flag resin respectively. Anti-Flag antibodies were used to analyse the presence of Flag-tagged proteins.

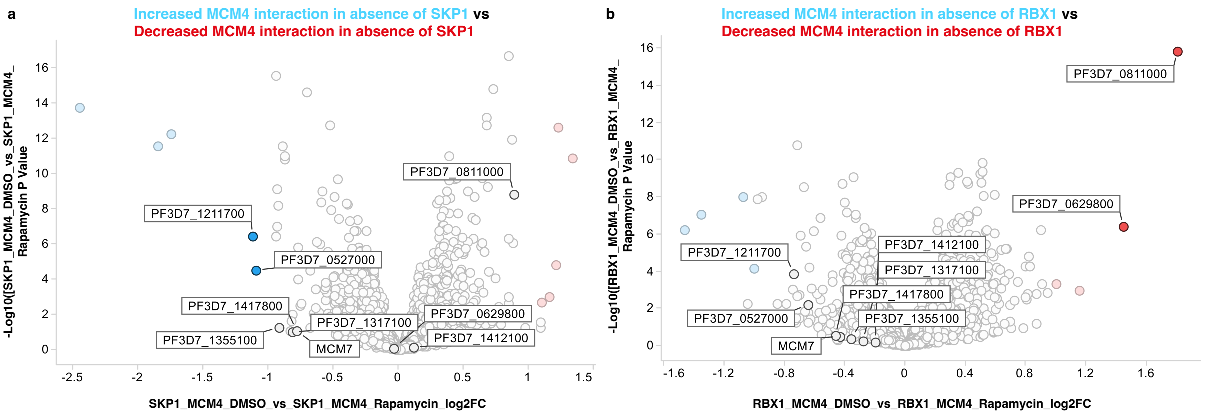

Supplementary Figure 6. Analysis of MCM4 interacting proteins in PfRBX1/PfSKP1 deleted parasites. a. Volcano plot of LC-MS/MS analysis of anti-Flag immunoprecipitation in PfMCM4-Flag containing DiCre-SKP1 strain parasites, treated with DMSO or rapamycin. >2-fold increased or decreased PfMCM4 interacting proteins in immunoprecipitate marked with red and blue circles respectively. Proteins of interest to this study are further labelled with protein names. b. Volcano plot of LC-MS/MS analysis of anti-Flag immunoprecipitation in PfMCM4-Flag containing DiCre-RBX1 strain parasites, treated with DMSO or rapamycin. >2-fold increased or decreased PfMCM4 interacting proteins in immunoprecipitate marked with red and blue circles respectively. Proteins of interest to this study are further labelled with protein names.

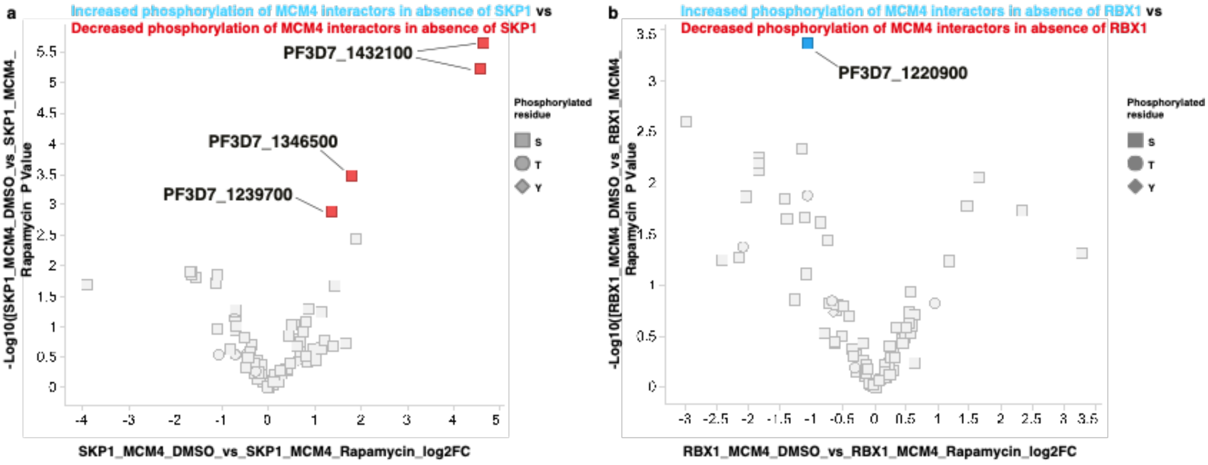

Supplementary Figure 7. Analysis of phosphopeptides of MCM4 interacting proteins in PfRBX1/PfSKP1 deleted parasites. a. Volcano plot of LC-MS/MS analysis of phosphorylated residues identified by anti-Flag immunoprecipitation in PfMCM4-Flag containing DiCre-SKP1 strain parasites, treated with DMSO or rapamycin. >2-fold increased or decreased peptides of PfMCM4 interacting proteins in immunoprecipitate marked with red and blue respectively. b. Volcano plot of LC-MS/MS analysis of phosphorylated residues identified by

anti-Flag immunoprecipitation in PfMCM4-Flag containing DiCre-RBX1 strain parasites, treated with DMSO or rapamycin. >2-fold increased or decreased peptides of PfMCM4 interacting proteins in immunoprecipitate marked with red and blue respectively.

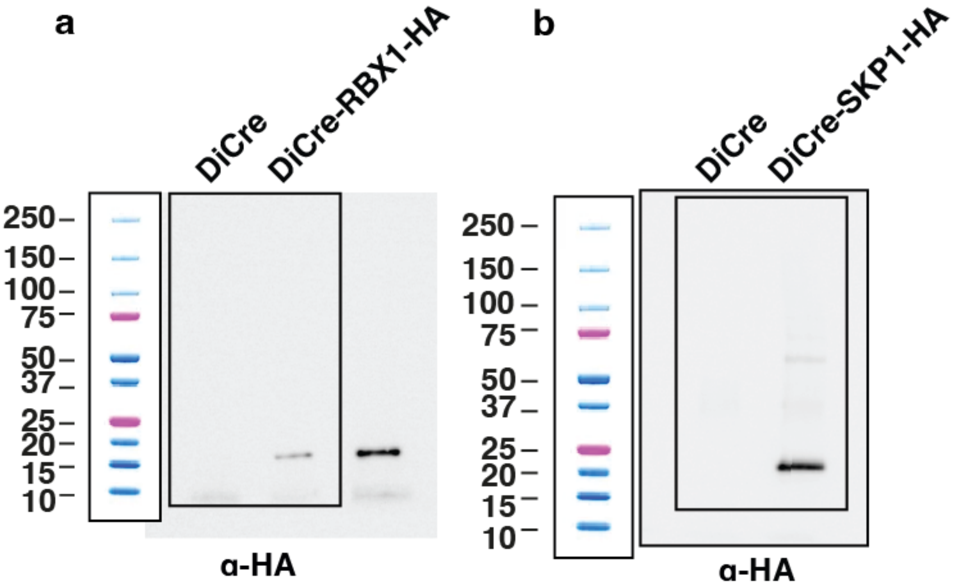

Supplementary Figure 8. Uncropped western blots from Figure 1a and 1b. a. Boxes display cropped regions for figure 1a.

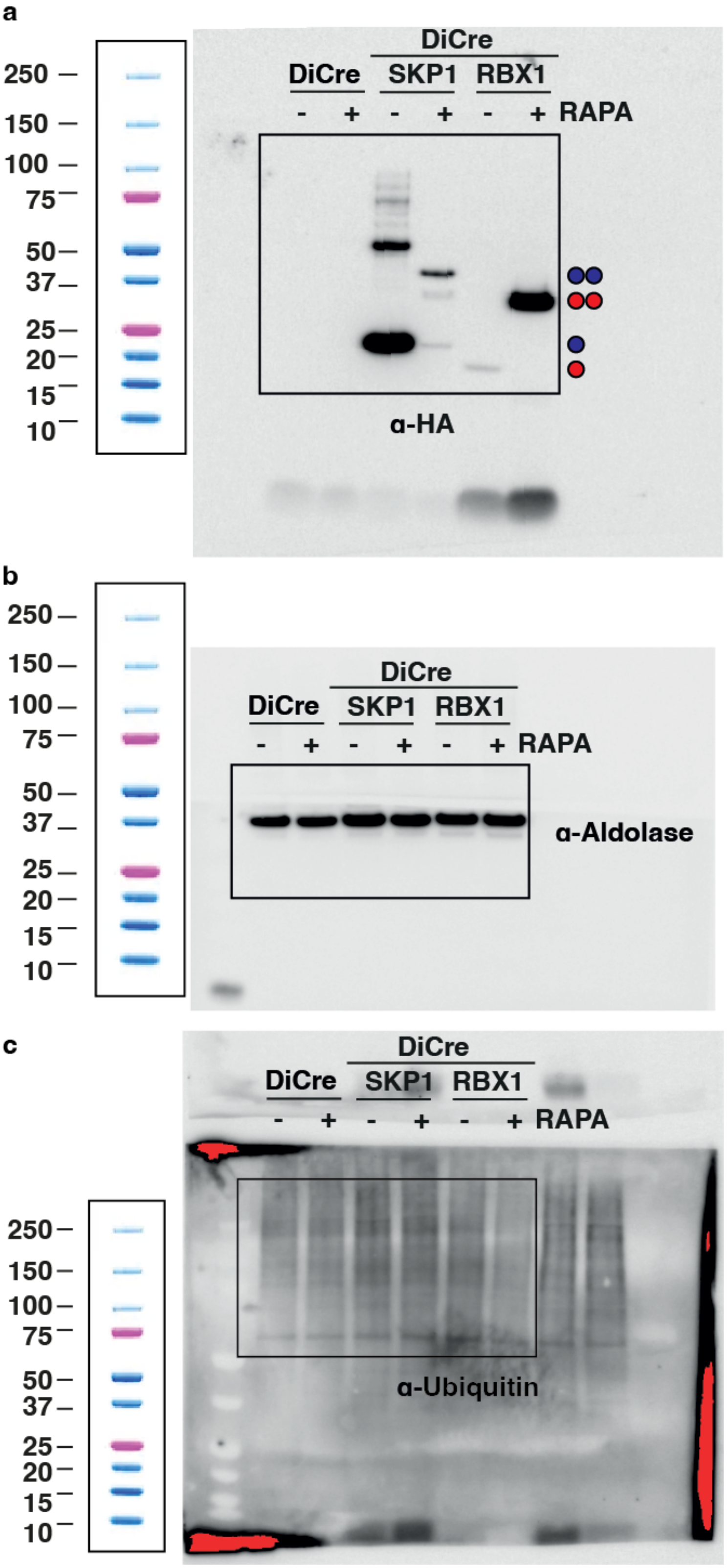

Supplementary Figure 9. Uncropped western blots from Figure 2c. a-c. Boxes display cropped regions for figure 2c.

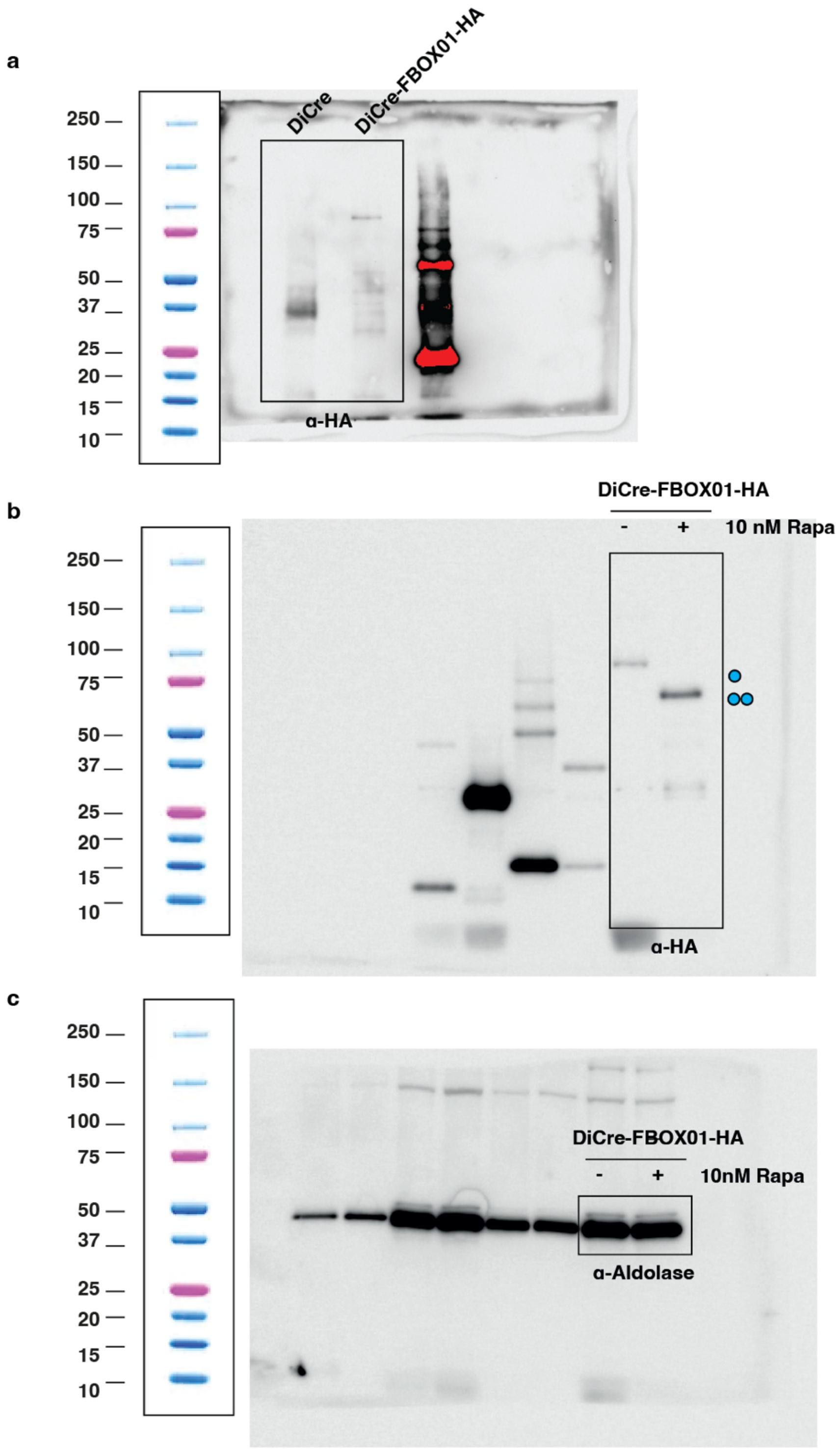

Supplementary Figure 10. Uncropped western blots from Figure 4a and c. a. full western blot corresponding to figure 4a with the boxed region referring to the cropped area. b. full western blot corresponding to figure 4c with the boxed region referring to the cropped area. c. full western blot corresponding to figure 4c with the boxed region referring to the cropped area.

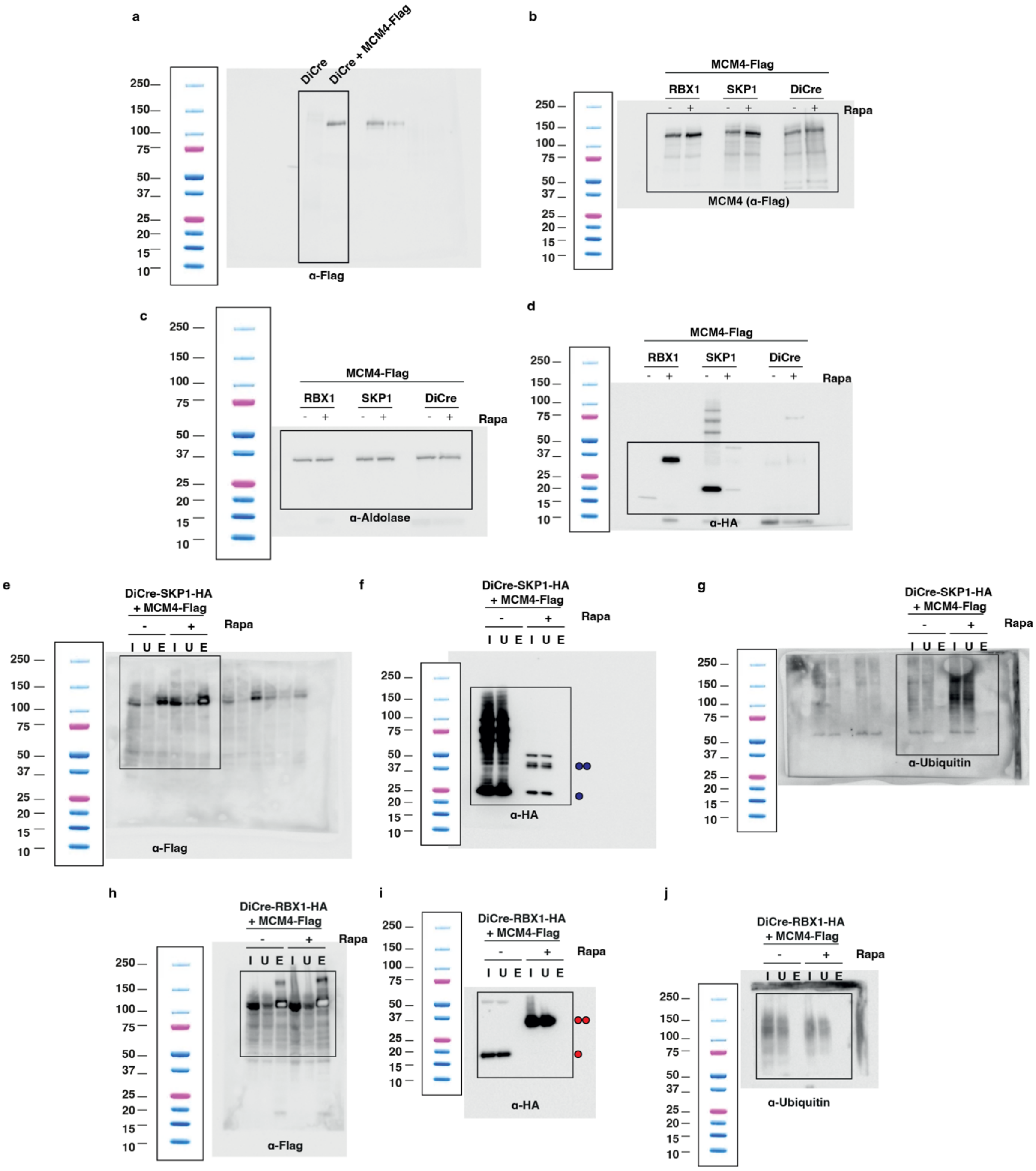

Supplementary Figure 11. Uncropped western blots from Figure 5a,5c,5d and 5e. a. Boxes display cropped regions for figure 5a. b, c & d. Boxes display cropped regions for figure 5c. e, f & g. Boxes display cropped regions for figure 5d. h, I & j. Boxes display cropped regions for figure 5e

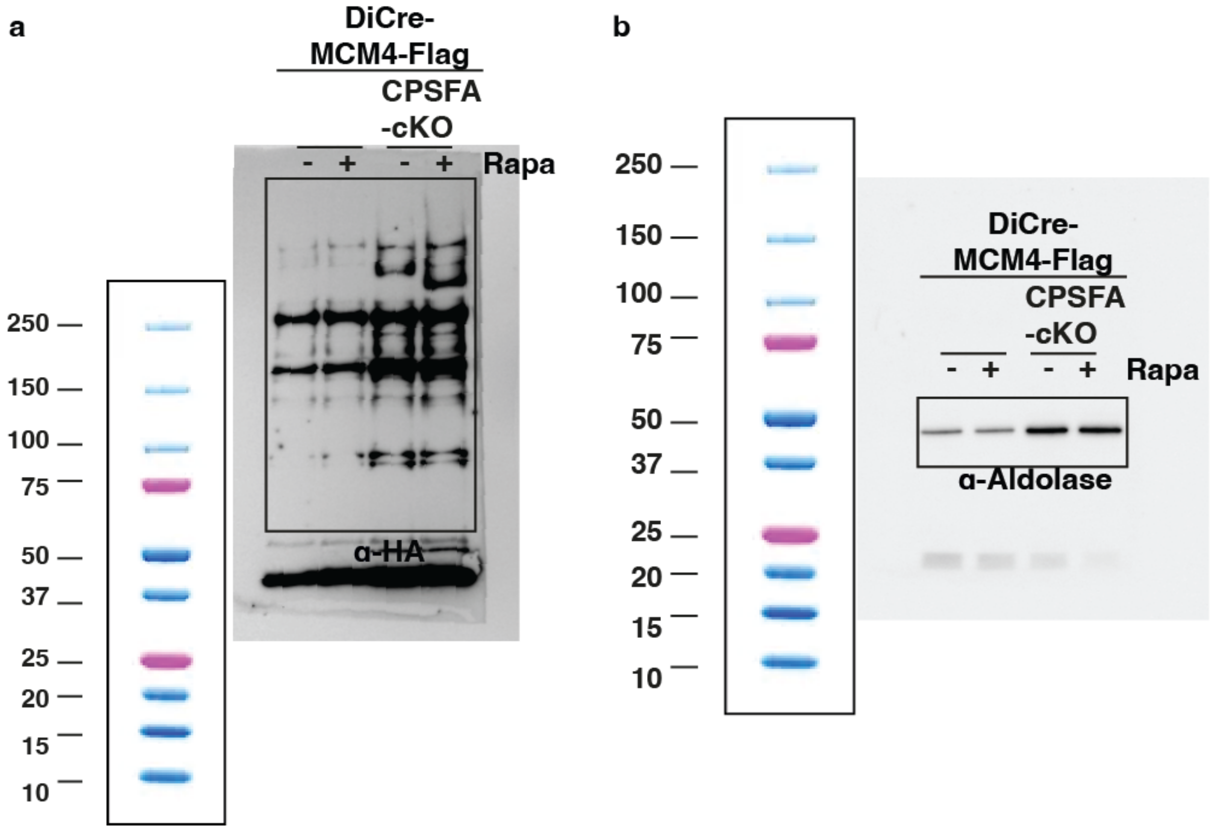

Supplementary Figure 12. Uncropped western blots from Figure 6c. a & b. Boxes display cropped regions for figure 6c.

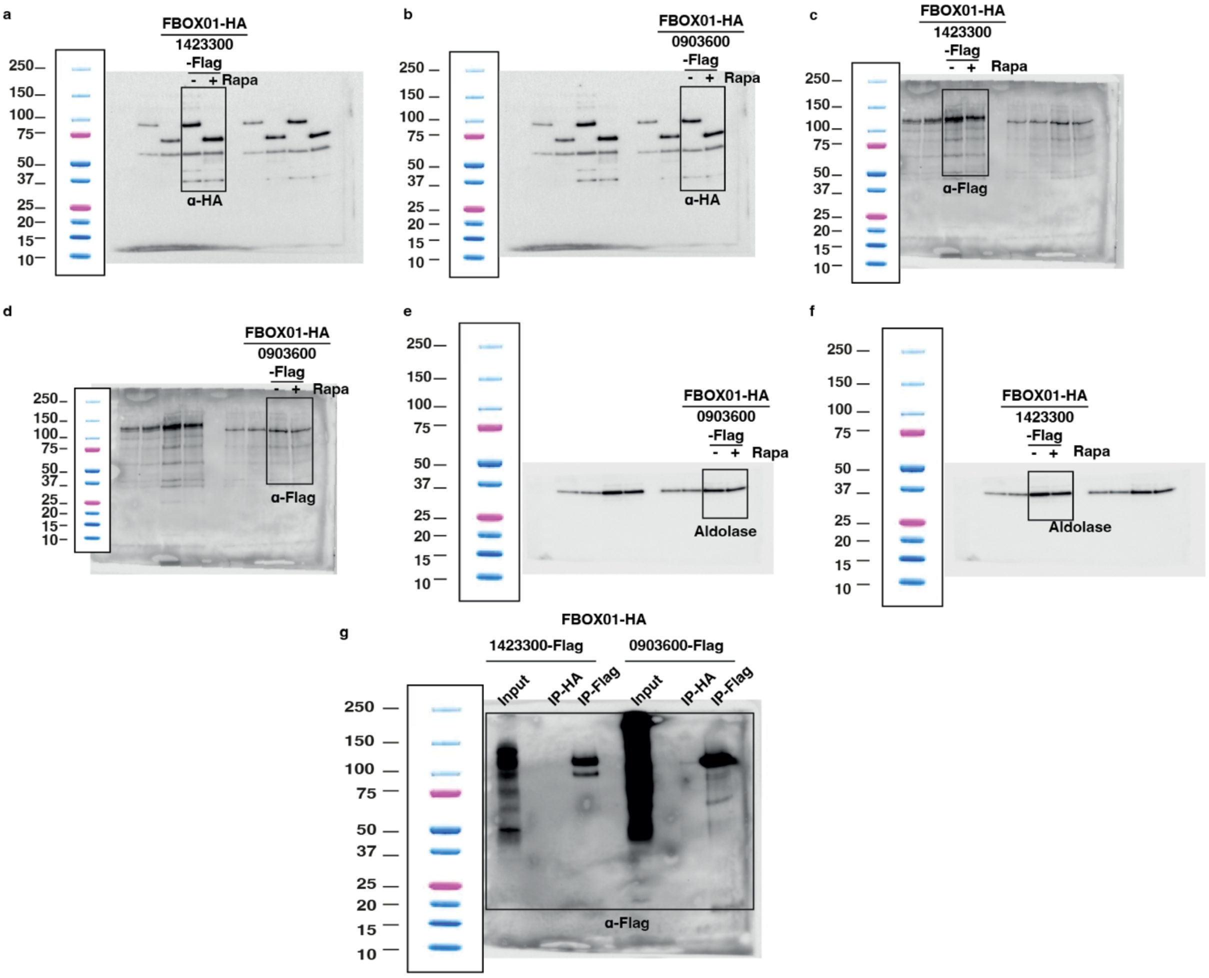

Supplementary Figure 13. Uncropped western blots from Supplementary Figure 5b and 5c. a, b, c, d, e & f. Boxes display cropped regions for figure S5b. g. Boxes display cropped regions for figure 5c.
